# Cycloalkyl Groups as Building Blocks of Artificial Carbohydrate Receptors: Studies with Macrocycles Bearing Flexible Side-Arms

**DOI:** 10.3390/molecules27217630

**Published:** 2022-11-07

**Authors:** Betty Leibiger, Manuel Stapf, Monika Mazik

**Affiliations:** Institut für Organische Chemie, Technische Universität Bergakademie Freiberg, Leipziger Straße 29, 09596 Freiberg, Germany

**Keywords:** molecular recognition, macrocycle, cycloalkyl groups, carbohydrates, van der Waals interactions, receptor

## Abstract

The cyclopentyl group was expected to act as a building block for artificial carbohydrate receptors and to participate in van der Waals contacts with the carbohydrate substrate in a similar way as observed for the pyrrolidine ring of proline in the crystal structures of protein-carbohydrate complexes. Systematic binding studies with a series of 1,3,5-trisubstituted 2,4,6-triethylbenzenes bearing various cycloalkyl groups as recognition units provided indications of the involvement of these groups in the complexation process and showed the influence of the ring size on the receptor efficiency. Representatives of compounds that exhibit a macrocyclic backbone and flexible side arms were now chosen as further model systems to investigate whether the previously observed effects represent a general trend. Binding studies with these macrocycles towards *β*-D-glucopyranoside, an all-equatorial substituted carbohydrate substrate, included ^1^H NMR spectroscopic titrations and microcalorimetric investigations. The performed studies confirmed the previously observed tendency and showed that the compound bearing cyclohexyl groups displays the best binding properties.

## 1. Introduction

Despite many interesting and promising results achieved in recent years, the selective and exactly predictable recognition of carbohydrates by artificial receptors employing noncovalent interactions remains a challenge in supramolecular chemistry (for examples of reviews, see refs. [1,2,3,4,5,6,7,8,9,10,11]; for recent reports on binding studies, see refs. [12,13,14,15,16,17,18,19,20,21,22,23,24,25,26,27,28,29]). On the one hand, such model systems help to better understand the phenomena of biological carbohydrate-mediated processes, and on the other hand, X-ray structural analyses of the protein-carbohydrate complexes [30,31,32,33,34,35,36,37,38,39,40,41,42] serve as a source of ideas for the design of the biomimetic systems. The driving force behind such studies is, among others, the potentially important role of the carbohydrate-binding agents in the development of novel therapeutics and/or diagnostics (e.g., anti-infectives or cancer diagnostic agents) [43,44,45,46,47].

**Figure 1:**
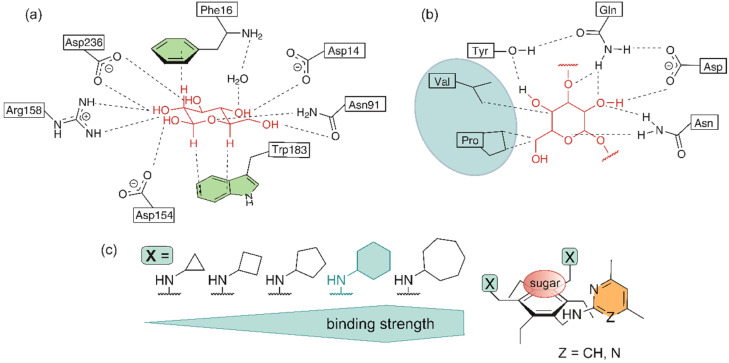
Examples of (**a**) neutral and charge-enhanced hydrogen bonds as well as CH···π interactions in the complex of D-galactose-binding protein (GBP) with d-glucose (adapted with permission from ref. [40], copyright 1988 AAAS) and (**b**) hydrogen bonds and van der Waals contacts in the complex of *Narcissus pseudonarcissus* lectin with Man(α1–3)Man (adapted with permission from ref. [42], copyright 1999 Elsevier). (**c**) Schematic illustration of acyclic compounds bearing cycloalkyl groups as recognition units (binding strength towards octyl *β*-D-glucopyranoside) [27].

Inspired by the interactions that are responsible for the selective recognition of carbohydrates by proteins (see Figure 1a,b), artificial carbohydrate-binding compounds with the capability to complex the carbohydrate substrate by a combination of hydrogen bonding, CH∙∙∙π interactions and numerous van der Waals contacts have been developed. The participation of the side chains of valine, leucine and proline in van der Waals contacts with the sugar substrate (Figure 1b) [41,42] has inspired us, for example, to use the isopropyl, isobutyl and cycloalkyl groups as subunits of artificial receptor molecules [27,48]. Systematic binding studies with a series of 1,3,5-trisubstituted 2,4,6-triethylbenzenes have provided clear indications of the participation of the aforementioned groups in the complexation process and also showed the influence of the ring size of the cycloalkyl groups on the receptor efficiency [27].

**Figure 2:**
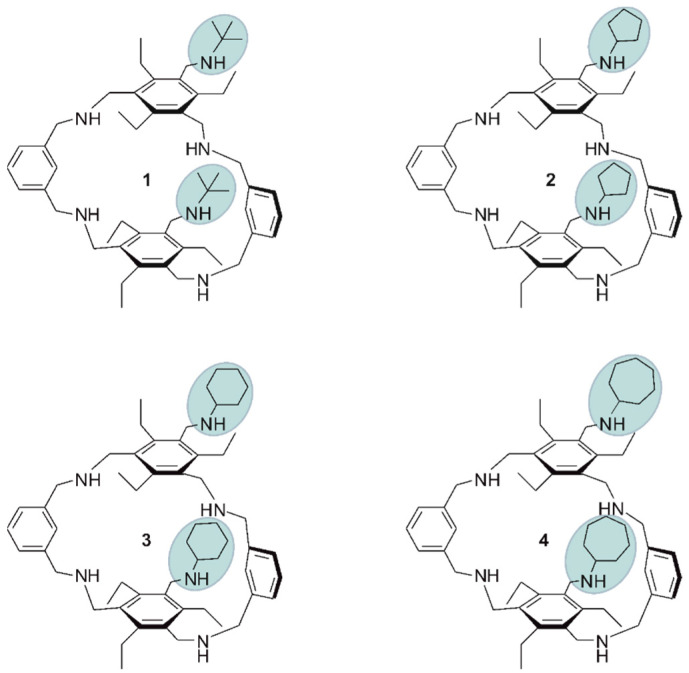
Structures of the macrocyclic target compounds **1**–**4** with flexible side arms bearing *tert*-butyl and cycloalkyl groups.

The aim of the present study was to investigate whether the previously observed involvement of the (cyclo)alkyl groups in the complexation process and the influence of ring size on receptor affinity represent a general tendency. Representatives of compounds possessing a macrocyclic backbone and two flexible side arms were chosen as further model systems (see Figure 2). The nature of the side-arms was previously shown to have a significant effect on the binding properties of compounds belonging to this receptor class.

Such receptor architecture was inspired by the results of our binding studies with acyclic receptor molecules, especially by the crystallographic studies. The design of this receptor type is briefly described below (see Section 2.1).

## 2. Results and Discussion

### 2.1. Design Principle and Selection Criteria: From Acyclic Receptors to Macrocycles with Flexible Side-Arms

**Figure 3:**
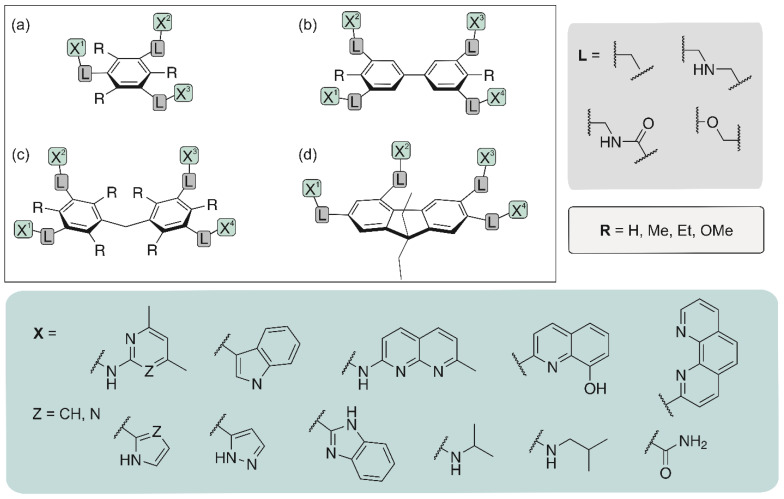
Examples of acyclic receptor molecules studied by our group: benzene (**a**), biphenyl (**b**), diphenylmethane (**c**) and fluorene (**d**) derivatives.

In recent years we have developed a wide range of acyclic [8,9,17,27,48,49,50,51,52,53,54,55,56,57,58,59,60] and macrocyclic carbohydrate receptors [13,61,62,63]. Among the acyclic receptors, compounds with a central benzene core as well as with a biphenyl or diphenylmethane [49,52,54] scaffold have been investigated (for examples, see Figure 3). In addition, fluorene derivatives have been considered as potential carbohydrate receptors [64]. Depending on the nature of the recognition groups (units X in Figure 3) and the way of their connection with the aromatic platform, carbohydrate receptors with different binding properties could be obtained.

Such compounds are relatively easy to prepare and the acyclic architecture is particularly well suited for systematic variations, but the formation of complexes with higher stoichiometry is in some cases a disadvantage. However, our studies have demonstrated that the binding properties of this type of compounds can be fine-tuned by variation of the receptor subunits, as confirmed, for example, in the case of compounds with a purine moiety as a building block [17].

**Figure 4:**
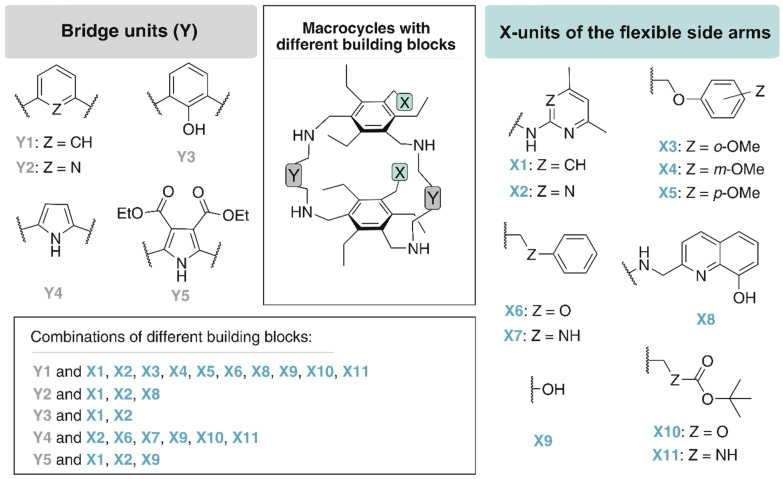
Examples of building blocks used by our group for the construction of macrocycles bearing two flexible side-arms.

By combining a macrocyclic backbone with flexible side arms, as illustrated in Figure 4, receptor molecules have been developed that predictably form 1:1 complexes with some carbohydrate substrates.

**Figure 5:**
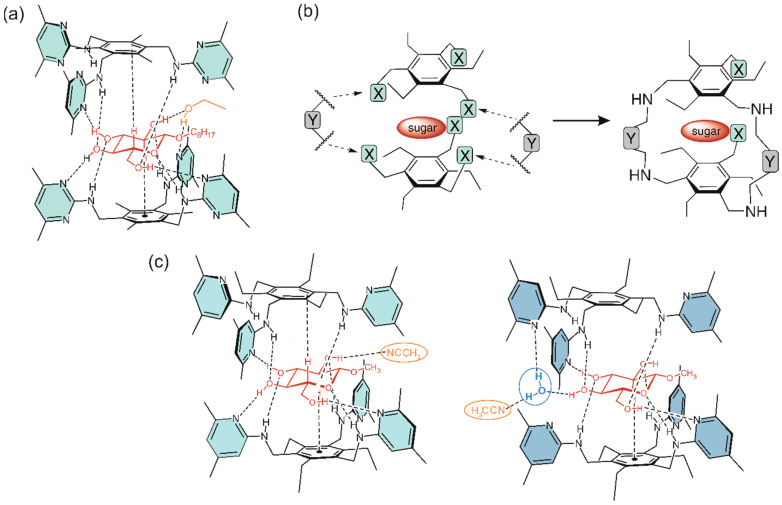
Schematic representation of hydrogen bonds and C-H∙∙∙π interactions in a crystalline 2:1 receptor-glucopyranoside complex (adapted with permission from ref. [60], copyright 2005 American Chemical Society) (**a**), which inspired the development of macrocyclic receptors with two flexible side-arms, as shown in (**b**), as well as the schematic representation of other 2:1 receptor-glucopyranoside complexes published by our group (**c**) [19]. The structural variation of the macrocyclic compounds, the structure of which is shown schematically in (**b**), included the variation of the bridging moieties (units Y) and the functional groups of the side-arms (units X).

The design of macrocycles with two flexible side-arms (see Figure 5b) was mainly inspired by the crystal structure of the 2:1 receptor-glucopyranoside complex [60] shown in Figure 5a. Structural variations, which included the incorporation of different bridging units (moieties Y) and the variation of the functional groups of the side-arms (units X), resulted in a number of new molecules (see Figure 4) [13,61,62,63].

In addition to the crystalline complex shown in Figure 5a, crystal structures of other 2:1 receptor-sugar complexes were obtained (Figure 5c), [18,19] confirming the binding motif observed in the first crystal structure. The carbohydrate is embedded between two benzene rings, thus forming CH∙∙∙π interactions with these central aromatic moieties of the two receptor molecules (for examples of other macrocyclic carbohydrate receptors, see refs. [1,2,3,4,5,6,7,12,65,66,67,68]). All-equatorially substituted sugars, such as *β*-glucopyranosides, are particularly well suited to participate in the formation of the 1:1 complexes, therefore octyl *β*-D-glucopyranoside (**βGlc**) was chosen as the model substrate in these studies. 

The extensive studies already performed allowed the identification of interesting structure-binding activity relationships, which are very useful for further developments in this research area. For the current studies, the phenylene-bridged macrocycles were chosen (see Figure 2), in which each of the two (cyclo)alkyl groups is incorporated into the receptor structure via an NH unit. The presence of a hydrogen bond donor site in the flexible side-arms of the macrocyclic receptors was previously shown to be necessary to achieve effective carbohydrate binding. Even in the presence of a sterically demanding residue, [13] the adjacent hydrogen bond donor was shown to contribute to effective complexation of the carbohydrate substrate.

### 2.2. Synthesis of the Target Compounds

**Scheme 1:**
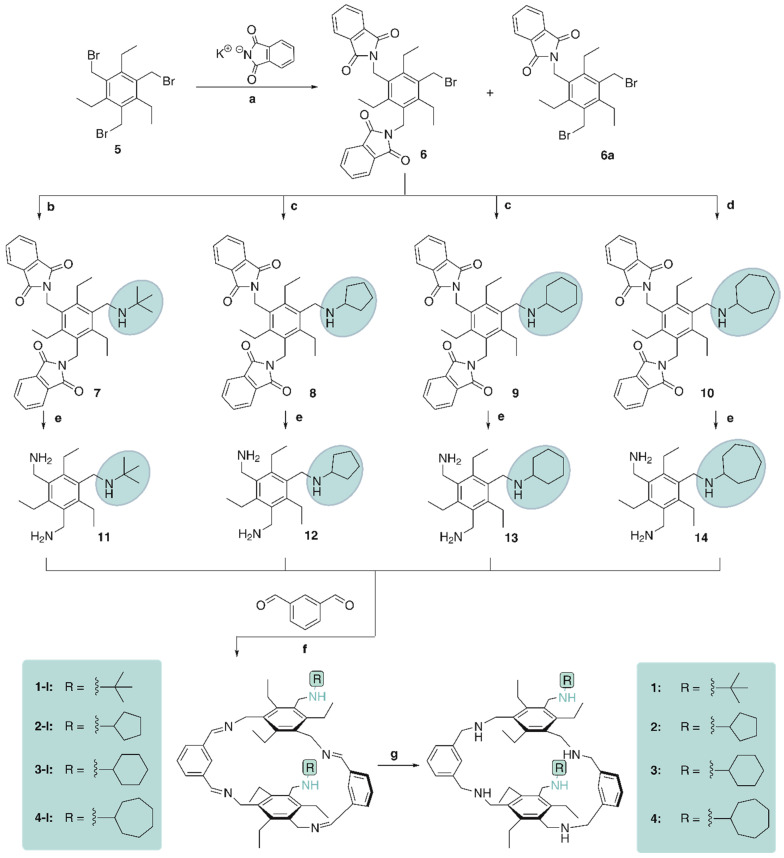
Reaction conditions: (**a**) potassium phthalimide (2 equiv.), DMF/dioxane; (**b**) *tert*-butylamine (2.5 equiv.), triethylamine, CH_3_CN/THF; (**c**) cyclopentylamine or cyclohexylamine (1.5 equiv.), triethylamine, CH_3_CN/THF; (**d**) cycloheptylamine (2.0 equiv.), triethylamine, CH_3_CN/THF; (**e**) hydrazine hydrate (N_2_H_4_·H_2_O), EtOH/ toluene; (**f**) EtOH, isophthalaldehyde (1 equiv.); (**g**) NaBH_4_, MeOH/CH_2_Cl_2_.

For the synthesis of the target compounds **1**–**4**, the commonly known precursor 1,3,5-tris(bromomethyl)-2,4,6-triethylbenzene (**5**) was used, which was prepared from commercially available 1,3,5-triethylbenzene [69]. By reacting **5** with two equivalents of potassium phthalimide in a solvent mixture of *N*,*N*-dimethylformamide and 1,4-dioxane, the derivative **6**, bearing two phthalimidomethyl groups, and the by-product **6a** were obtained (see Scheme 1). The separation of compound **6** was performed by column chromatography. Crystallization of **6** from toluene yields crystals of the monoclinic space group *P*2_1_/*n* with one molecule in the asymmetric unit of the cell, as described in ref. [70].

Compound **6** was converted into the (cyclo)alkylamino-substituted benzene derivatives **7**–**10** by reaction with the corresponding primary amine, such as *tert*-butylamine, cyclopentylamine, cyclohexylamine or cycloheptylamine. The reaction was carried out using triethylamine as base in a solvent mixture of tetrahydrofuran and acetonitrile. By using microwave irradiation (150 W) at 100 °C, the reaction required only 10 min. The required equivalents of primary amine depended on the steric demand and reactivity of the educts (see Section 4). Subsequently, compounds **7**–**10** were converted into the corresponding bis(aminomethyl)-substituted benzene derivatives **11**–**14** by hydrazinolysis in toluene and ethanol. 

In the macrocyclization reactions, compounds **11**–**14** reacted stoichiometrically with commercially available isophthalaldehyde in ethanol at 40–50 °C. The macrocyclic imines **1-I**–**4-I** precipitated as white solids and were separated by centrifugation. Their reduction with sodium borohydride in a solvent mixture of methanol and dichloromethane yielded the target compounds **1**–**4**. Starting from precursors **11**–**14**, the yields over two steps of **1**–**4** ranged between 50 and 65%.

### 2.3. Binding Studies

The complexation properties of compounds **1**–**4** towards the selected carbohydrate substrate, octyl *β*-d-glucopyranoside (**βGlc**), were evaluated on the basis of ^1^H NMR spectroscopic titrations and isothermal titration calorimetry (ITC). In addition, 2D NMR investigations were carried out and the experimental studies were supported by molecular modelling calculations.

#### 2.3.1. ^1^H NMR Titrations

**Figure 6:**
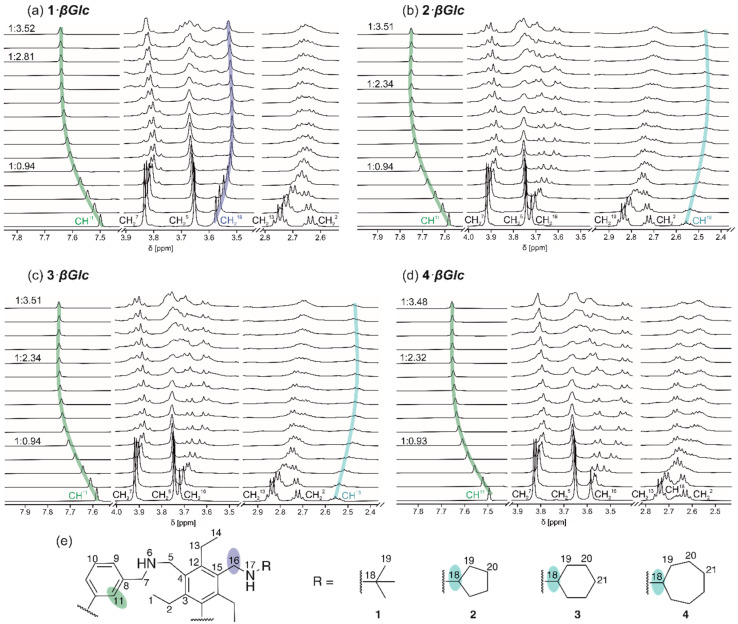
Partial ^1^H NMR spectra (500 MHz, CDCl_3_, 293 K) of the compounds (**a**) **1**, (**b**) **2**, (**c**) **3** and (**d**) **4** after addition of (**a**) 0.00–3.52 equiv. of **βGlc** ([**1**] = 1.00 mM), (**b**) 0.00–3.51 equiv. of **βGlc** ([**2**] = 1.02 mM), (**c**) 0.00–3.51 equiv. of **βGlc** ([**3**] = 1.01 mM), (**d**) 0.00–3.48 equiv. of **βGlc** ([**4**] = 1.00 mM). For labelling, see the structure excerpts in (**e**).

The ^1^H NMR spectroscopic titrations were performed either with constant receptor concentration and increasing concentration of glucopyranoside(for examples, see Table S1) or with constant sugar concentration and variable receptor concentration (inverse titration) in CDCl_3_ at 293 K. The programs WinEQNMR [71] and SupraFit [72] as well as the mole ratio method were used to analyze the ^1^H NMR titration data. The determined binding constants are given in Table 1. Exemplary complexation-induced shifts observed for receptors **1**–**4** during titration with octyl *β*-D-glucopyranoside are shown in Figure 6 (see also Figures S1 and S2).

In all cases, the largest chemical shift change was observed for the aromatic CH^11^ signals of the macrocycles, which shifted all downfield. Furthermore, an upfield shift of the CH^18^ signals of compounds **2**–**4** was observed, indicating that the side-arms bearing cycloalkylamino groups (-CH_2_NHR) are involved in the complexation process. For the CH_2_^16^ signals of **1**–**4** always a significant upfield shift was observed.

All complexes show a fast exchange on the NMR time scale, which was also observed for some compounds of this type that we have previously studied (e.g., compounds displaying following combinations of the building blocks: Y1 and X8; Y2 and X1, X2 or X8; Y3 and X2, X4 or X10; see Figure 4). In addition to the complexes showing fast exchange on the NMR time scale, slow exchange was observed for some representatives of this class of compounds that have been previously tested (e.g., compounds with following combinations of the building blocks: Y4 and X2 or X11; Y5 and X2) [13,61,62,63].

Inverse titrations showed a downfield shift and very strong broadening of the OH proton signals of **βGlc**, indicating their involvement in the formation of hydrogen bonds. Furthermore, the upfield shifts of all sugar CH signals indicate the participation of these groups in CH···π interactions [73,74,75,76,77,78] with the central benzene rings of the corresponding receptor molecule.

The performed NMR studies revealed the ability of the macrocyclic compounds **1**–**4** to act as carbohydrate-binding agents (artificial carbohydrate receptors) and confirmed the formation of the 1:1 receptor-carbohydrate complexes in which the selected substrate is complexed by a combination of hydrogen bonds, CH···π interactions and van der Waals contacts. Although the differences between the binding strengths of these compounds are not large, a trend is recognizable. This trend was also fully confirmed by the microcalorimetric experiments, as described below (see Section 2.3.2).

Regarding the influence of the ring size on the binding strength of the receptor, both measurement methods reveal the same trend as that observed for the previously studied acyclic receptors [27]. Again, the incorporation of the cyclohexyl groups into the receptor structure gives better results than incorporation of the cyclopentyl or cycloheptyl groups, with the cycloheptyl-containing receptor being somewhat weaker than that bearing cyclopentyl groups. The receptor efficiency increases slightly in the order **4** < **2** ≤ **3** (i.e., cycloheptyl < cyclopentyl ≤ cyclohexyl group). According to the results of the two methods, compound **1** bearing *tert*-butylamino groups is a weaker receptor than that containing cycloalkyl groups.

**Figure 7:**
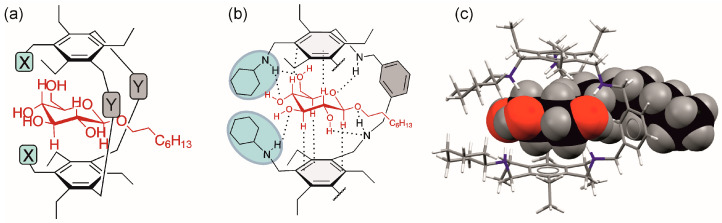
(**a**,**b**) Schematic illustration of the location of octyl *β*-d-glucopyranoside in the cavity of the macrocyclic receptor, as indicated by ^1^H NMR titrations and ROESY experiments. (**c**) Energy-minimized structure of the 1:1 complex of compound **3** and octyl *β*-D-glucopyranoside (**βGlc**). The sugar molecule is shown in the space-filling model. C atoms of the sugar: black, O atoms of the sugar: red, H atoms of the sugar: grey; C atoms of the receptor: grey, N atoms of the receptor: blue, H atoms of the receptor: white (Maestro 11.0, OPLS_2005 force field, MCMM, 50,000 steps).

Further structural information about the complexes formed was obtained by 2D NMR experiments (ROESY experiments; see Figures S6 and S7 in the Supplementary Materials), indicating that the glucopyranoside is placed in the receptor cavity, as schematically shown in Figure 7a, and is involved in the formation of the intermolecular interactions shown in Figure 7b. This binding mode was also confirmed by molecular modelling calculations (Figure 7c and Figure S5).

At this point it is important to note the excellent discussions on dispersive interactions reported by S. Kubik [79] and H.-J. Schneider [80]. As mentioned above, the use of cycloalkyl groups in the construction of carbohydrate receptors was inspired by the binding modes observed in protein-carbohydrate complexes, in particular by the involvement of the pyrrolidine ring of proline in the intermolecular interactions. In this context, it should be noted that the use of proline in the construction of some carbohydrate receptors has also been reported in the literature, as in the case of the effective cyclic hexapeptides composed of alternating subunits of L-proline and 3-aminobenzoic acid [81].

#### 2.3.2. Microcalorimetric Titrations

**Figure 8:**
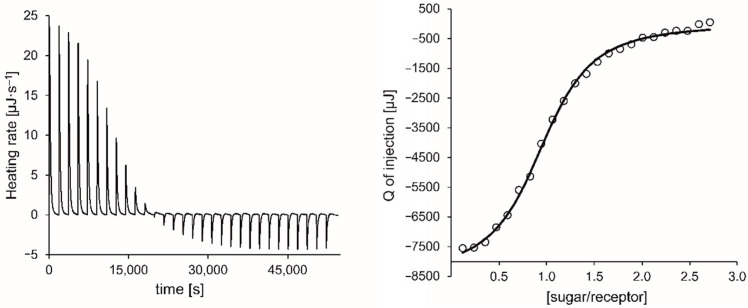
Exemplary ITC thermogram (**left**) and titration curve-fitting (**right**) for the titration of **3** with **βGlc** in CHCl_3_ at 20 °C (heat of dilution was subtracted).

Microcalorimetric titration experiments (isothermal titration calorimetry, ITC) were performed in CHCl_3_ or CHCl_3_/H_2_O (0.035 M H_2_O) by adding increasing amounts of octyl *β*-d-glucopyranoside (**βGlc**) to a solution of the corresponding receptor. At least three independent titration experiments were performed for each receptor/sugar pair. The data obtained were analyzed using the NanoAnalyze program (version 3.12.0 Copyright 2008, 2021 TA Instruments); the results are summarized in Table 2 (for comparison of the determined binding constants with those obtained by ^1^H NMR titrations, see Table 1; see also Figure 8 and Figures S3 and S4 in the Supplementary Materials). 

As stated above, the ITC results fully confirmed the trend indicated by the ^1^H NMR titrations in terms of binding strength. Accordingly, compound **3** with cyclohexylamino groups proved to be the one with the best binding properties under the chosen experimental conditions, although the differences between the binding strength of all investigated compounds were not very pronounced. The analyses showed an enthalpic driving force for all the complexation processes studied, which is partially compensated by the negative entropy. Such enthalpy-entropy compensation is well known and discussed in various literature sources [82,83,84,85].

In the case of compound **3**, the microcalorimetric titrations were also performed in the presence of small amounts of water. Under these experimental conditions, a significant increase in the binding strength of compound **3** was observed. One reason for these results could be the formation of water-mediated hydrogen bonds that favourable affect the complexation process. This is in agreement with observations on protein-carbohydrate complexes [36,38,40] and with results obtained with some artificial receptors [60,86,87]. For example, in the case of porphyrine-based receptors, the authors pointed out that the addition of water increase pyranoside binding “by filling in the gaps between the receptor and ligand” [86].

## 3. Conclusions

New representatives of the class of compounds containing both a macrocyclic backbone and two flexible side-arms were prepared, and their ability to act as carbohydrate receptors was evaluated by ^1^H NMR spectroscopic titrations and isothermal titration calorimetry experiments. The target compounds **1**–**4** were prepared via multi-step syntheses, which included the use of microwave-assisted reactions, among others. 

**Figure 9:**
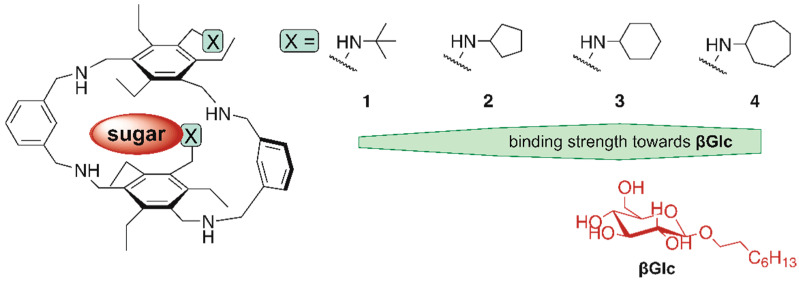
Schematic illustration of the binding strengths of compounds **1**–**4** towards **βGlc**.

Interestingly, both methods used in the binding studies confirmed the trend previously observed for the acyclic receptors and showed that the incorporation of the cyclohexyl groups in the receptor structure, as in **3**, has a more favourable effect on receptor efficiency than the other groups considered in the structural variations performed (see Figure 9), although the differences in binding affinities are not very pronounced.

Enthalpic driving force and enthalpy-entropy compensation were observed in all the binding studies performed. It should also be noted that in the case of compound **3**, the use of a water-containing organic solvent led to an increase in its binding strength under the selected experimental conditions.

Both the results of ^1^H NMR titrations and ROESY experiments indicated the formation of 1:1 complexes stabilized by hydrogen bonding, CH∙∙∙π and van der Waals interactions, thus confirming the expectations.

## 4. Experimental Section

Analytical TLC was carried out on pre-coated TLC sheets with 0.20 mm silica gel with fluorescent indicator UV_254_. For column chromatography, an Isolera^TM^ system (biotage) with silica gel columns was used. A Discover SP microwave reactor (CEM) was used for microwave-assisted syntheses. The synthesis of **6** is described in ref. [70]. 1H and 13C NMR spectra of compounds **1–4**, **1-I–4-I** and **7–10** are given in Figures S8–S31 (see Supplementary Materials).

Descriptions of the binding studies (^1^H NMR spectroscopic titrations and microcalorimetric titrations) are given in the Supplementary Materials. Octyl *β*-d-glucopyranoside is commercially available (Carbosynth, purity ≥ 99.5%).

*General procedure for the synthesis of compounds **7**–**10***

1,3-Bis(phthalimidomethyl)-5-(brommethyl)-2,4,6-triethylbenzene (**6**) dissolved in a CH_3_CN/THF mixture (24 mL, 2:1, *v*/*v*) was placed in a 35 mL microwave vessel and the equimolar amount of triethylamine as well as an excess of the corresponding primary amine were added. The reaction mixture was heated to 100 °C and irradiated with microwaves for 10 min (mode: fixed power, 150 W; CEM Discover SP microwave reactor). Then, the solvent was removed under reduced pressure and the resulting crude product was separated by flash chromatography (toluene/ethyl acetate, 6 → 66% ethyl acetate over 15 column volumes, CV). The products **7**–**10** were obtained as white solids.

*1,3-Bis(phthalimidomethyl)-5-tert-butylaminomethyl-2,4,6-triethylbenzene* (**7**). Compound **7** was prepared from **6** (500 mg, 0.87 mmol) and *tert*-butylamine (228 μL, 2.18 mmol). Yield 75% (367 mg, 0.65 mmol); R_f_ = 0.13 [toluene/ethyl acetate, 2:1 (*v*/*v*)]; m.p. 228 °C; ^1^H NMR (500 MHz, CDCl_3_): *δ* = 0.94 (t, 3H, *J* = 7.6 Hz), 1.08 (t, 6H, *J* = 7.5 Hz), 1.14 (s, 9H), 2.89 (q, 4H, *J* = 7.5 Hz), 3.19 (q, 2H, *J* = 7.6 Hz), 3.65 (s, 2H), 4.93 (s, 4H), 7.65–7.69 (m, 4H), 7.77–7.81 (m, 4H) ppm; ^13^C NMR (125 MHz, CDCl_3_): *δ* = 16.1, 16.3, 22.8, 23.5, 28.9, 37.6, 40.1, 50.5, 123.3, 129.4, 132.2, 134.0, 135.1, 144.4, 144.6, 168.4 ppm; HRMS (ESI): *m*/*z* calcd for C_35_H_39_N_3_O_4_+H^+^: 566.3013 [M+H]^+^, found: 566.3001.

*1,3-Bis(phthalimidomethyl)-5-cyclopentylaminomethyl-2,4,6-triethylbenzene* (**8**). Compound **8** was prepared from **6** (500 mg, 0.87 mmol) and cyclopentylamine (129 μL, 1.31 mmol). Yield 64% (322 mg, 0.56 mmol); R_f_ = 0.21 [toluene/ethyl acetate, 2:1 (*v*/*v*)]; m.p. 95 °C; ^1^H NMR (500 MHz, CDCl_3_): *δ* = 0.95 (t, 3H, *J* = 7.6 Hz), 1.08 (t, 6H, *J* = 7.6 Hz), 1.36–1.42 (m, 2H), 1.47–1.55 (m, 2H), 1.64–1.70 (m, 2H), 1.76–1.82 (m, 2H), 2.89 (q, 4H, *J* = 7.5 Hz), 3.15 (qui, 1H, *J* = 7.6 Hz), 3.18 (q, 2H, *J* = 7.6 Hz), 3.66 (s, 2H), 4.92 (s, 4H), 7.65–7.69 (m, 4H), 7.77–7.81 (m, 4H) ppm; ^13^C NMR (125 MHz, CDCl_3_): *δ* = 16.1, 16.3, 23.0, 23.5, 24.1, 33.1, 37.6, 46.5, 60.9, 123.3, 129.4, 132.2, 134.0, 135.0, 144.2, 144.8, 168.4 ppm; HRMS (ESI): *m*/*z* calcd for C_36_H_39_N_3_O_4_+H^+^: 578.3013 [M+H]^+^, found: 578.2990.

*1,3-Bis(phthalimidomethyl)-5-cyclohexylaminomethyl-2,4,6-triethylbenzene* (**9**). Compound **9** was prepared from **6** (500 mg, 0.87 mmol) and cyclohexylamine (150 μL, 1.31 mmol). Yield 79% (410 mg, 0.69 mmol); R_f_ = 0.12 [toluene/ethyl acetate, 2:1 (*v*/*v*)]; m.p. 102 °C; ^1^H NMR (500 MHz, CDCl_3_): *δ* = 0.95 (t, 3H, *J* = 7.6 Hz), 1.08 (t, 6H, *J* = 7.5 Hz), 1.13–1.30 (m, 5H), 1.57–1.60 (m, 1H), 1.69–1.73 (m, 2H), 1.87–1.90 (m, 2H), 2.49–2.52 (m, 1H), 2.88 (q, 4H, *J* = 7.5 Hz), 3.19 (q, 2H, *J* = 7.5 Hz), 3.70 (s, 2H), 4.93 (s, 4H), 7.65–7.69 (m, 4H), 7.77–7.81 (m, 4H) ppm; ^13^C NMR (125 MHz, CDCl_3_): *δ* = 16.0, 16.2, 22.8, 23.4, 24.9, 26.2, 33.4, 37.5, 44.9, 59.9, 123.2, 129.2, 132.0, 133.9, 135.0, 144.0, 144.6, 168.2 ppm; HRMS (ESI): *m*/*z* calcd for C_37_H_41_N_3_O_4_+H^+^: 592.3169 [M+H]^+^, found: 592.3147.

*1,3-Bis(phthalimidomethyl)-5-cycloheptylaminomethyl-2,4,6-triethylbenzene* (**10**). Compound **10** was prepared from **6** (500 mg, 0.87 mmol) and cycloheptylamine (222 μL, 1.74 mmol). Yield 86% (452 mg, 0.75 mmol); R_f_ = 0.30 [toluene/ethyl acetate, 2:1 (*v*/*v*)]; m.p. 182 °C; ^1^H NMR (500 MHz, CDCl_3_): *δ* = 0.96 (t, 3H, *J* = 7.6 Hz), 1.09 (t, 6H, *J* = 7.6 Hz), 1.37–1.45 (m, 4H), 1.47–1.57 (m, 4H), 1.61–1.67 (m, 2H), 1.80–1.85 (m, 2H), 2.69–2.74 (m, 1H), 2.87 (q, 4H, *J* = 7.5 Hz), 3.20 (q, 2H, *J* = 7.6 Hz), 3.65 (s, 2H), 4.93 (s, 4H), 7.65–7.69 (m, 4H), 7.77–7.81 (m, 4H) ppm; ^13^C NMR (125 MHz, CDCl_3_): *δ* = 16.1, 16.3, 22.9, 23.5, 24.3, 28.6, 34.9, 37.6, 45.8, 60.5, 123.3, 129.4, 132.1, 134.0, 135.1, 144.2, 144.8, 168.4 ppm; HRMS (ESI): *m*/*z* calcd for C_38_H_43_N_3_O_4_+H^+^: 606.3326 [M+H]^+^, found: 606.3311.

*General procedure for the synthesis of compounds **11**–**14***

Hydrazine hydrate (4.5 equiv.) was added to the corresponding bis(phthalimidomethyl)-substituted benzene derivative (**7**–**10**) dissolved in toluene/ethanol 2:1 (*v*/*v*) and the reaction mixture was stirred under reflux and argon atmosphere for 7 h. After allowing the reaction mixture to cool to room temperature, the solvents were removed under reduced pressure. The resulting residue was suspended in toluene and an aqueous KOH solution (40% *w*/*v*) was added until the white solid was completely dissolved. The organic phase was separated, washed with a little amount of brine and dried over Na_2_SO_4_. After removing the solvent under vacuum, the products **11**–**14** were obtained as colourless oils (**12**–**14**) or a white solid (**11**). The crude products were used directly for further reaction.

*General procedure for the synthesis of the macrocycles **1-I**–**4-I** and the target compounds **1**–**4***

Isophthalaldehyde was added to a solution of the bis(aminomethyl)-substituted benzene derivative **11**, **12**, **13** or **14** in dry ethanol and the resulting mixture was stirred at room temperature (the details for the individual reaction are given below). The precipitated macrocyclic imines were separated by centrifugation, washed with small amounts of ethanol, and dried in vacuum. The imines **1-I**–**4-I** were obtained as white solids.

The corresponding imine was dissolved in a mixture of methanol and dichloromethane 2:1 (*v*/*v*) and sodium borohydride was added slowly. The reaction mixture was stirred at room temperature (see below for details). Afterwards, the solvent was removed under reduced pressure and the residue was stirred in a water/chloroform-mixture [10 mL, 9:1 (*v*/*v*)] for another 24 h. The mixture was extracted with chloroform and the combined organic layers were dried over Na_2_SO_4_. After removal of the solvent under vacuum, the products **1**–**4** were obtained as white solids.

Compound **1-I** was prepared from **11** (153 mg, 0.50 mmol) and isophthalaldehyde (67 mg, 0.50 mmol) in 5 mL of dry ethanol. The mixture was stirred for 7 h at 50 °C. Yield 60% (121 mg, 0.15 mmol); m.p. 255 °C (decomp.); ^1^H NMR (500 MHz, CDCl_3_): *δ* = 1.15 (s, 18H), 1.16 (t, 6H, *J* = 7.6 Hz), 1.21 (t, 12H, *J* = 7.6 Hz), 2.42 (br, 4H), 2.64 (br, 8H), 3.67 (s, 4H), 4.98 (s, 8H), 7.46 (t, 2H, *J* = 7.7 Hz), 7.59 (br, 2H), 7.94 (d, 4H, *J* = 7.5 Hz), 7.99 (s, 4H) ppm; ^13^C NMR (125 MHz, CDCl_3_): *δ* = 15.8, 16.4, 22.9, 23.4, 28.9, 40.4, 50.6, 56.1, 129.0, 129.1, 129.8, 131.7, 135.5, 136.9, 143.0, 143.6, 159.5 ppm; HRMS (ESI): *m*/*z* calcd for C_54_H_74_N_6_+H^+^: 807.6048 [M+H]^+^, found: 807.6068.

Compound **2-I** was prepared from **12** (343 mg, 1.08 mmol) and isophthalaldehyde (147 mg, 1.09 mmol) in 10 mL of dry ethanol. The reaction mixture was stirred for 4.5 h at 50 °C. Yield 67% (300 mg, 0.36 mmol); m.p. 160 °C (decomp.); ^1^H NMR (500 MHz, CDCl_3_): *δ* = 1.20 (t, 6H, *J* = 7.5 Hz), 1.23 (t, 12H, *J* = 7.0 Hz), 1.36–1.41 (m, 4H), 1.48–1.54 (m, 4H), 1.63–1.70 (m, 4H), 1.78–1.86 (m, 4H), 2.40 (br, 4H), 2.64 (br, 8H), 3.16 (qui, 2H, *J* = 6.3 Hz), 3.68 (s, 4H), 4.98 (s, 8H), 7.46 (t, 2H, *J* = 7.8 Hz), 7.52 (br, 2H), 7.94 (d, 4H, *J* = 7.8 Hz), 7.95 (s, 4H) ppm; ^13^C NMR (125 MHz, CDCl_3_): *δ* = 15.9, 16.4, 23.1, 23.4, 24.1, 33.1, 46.9, 56.0, 61.0, 129.0 (2C), 129.8, 131.6, 135.3, 136.8, 143.2, 143.5, 159.4 ppm; HRMS (ESI): *m*/*z* calcd for C_56_H_74_N_6_+H^+^: 831.6048 [M+H]^+^, found: 831.6059.

Compound **3-I** was prepared from **13** (464 mg, 1.40 mmol) and isophthalaldehyde (188 mg, 1.40 mmol) in 13 mL of dry ethanol. The reaction mixture was stirred for 4.5 h at 50 °C. Yield 65% (389 mg, 0.45 mmol); m.p. 250 °C (decomp.); ^1^H NMR (500 MHz, CDCl_3_): *δ* = 1.10–1.33 (m, 10H), 1.17 (t, 6H, *J* = 7.5 Hz), 1.22 (t, 12H, *J* = 7.5 Hz), 1.59–1.63 (m, 2H), 1.71–1.75 (m, 4H), 1.91–1.93 (m, 4H), 2.42 (br, 4H), 2.50–2.55 (m, 2H), 2.65 (br, 8H), 3.74 (s, 4H), 4.99 (s, 8H), 7.47 (t, 2H, *J* = 7.8 Hz), 7.54 (br, 2H), 7.96 (d, 4H, *J* = 7.8 Hz), 7.97 (s, 4H) ppm; ^13^C NMR (125 MHz, CDCl_3_): *δ* = 15.7, 16.3, 23.0, 23.3, 25.9, 26.2, 33.5, 45.3, 55.9, 58.1, 128.9 (2C), 129.8, 131.6, 135.3, 136.7, 143.0, 143.3, 159.2 ppm; HRMS (ESI): *m*/*z* calcd for C_58_H_78_N_6_+H^+^: 859.6361 [M+H]^+^, found: 859.6365.

Compound **4-I** was prepared from **14** (230 mg, 0.67 mmol) and isophthalaldehyde (89 mg, 0.67 mmol) in 7 mL of dry ethanol. The reaction mixture was stirred for 7 h at 45 °C. Yield 66% (197 mg, 0.22 mmol); m. p. 258 °C (decomp.); ^1^H NMR (500 MHz, CDCl_3_): *δ* = 1.16 (t, 6H, *J* = 7.4 Hz), 1.21 (t, 12H, *J* = 7.4 Hz), 1.40–1.46 (m, 8H), 1.47–1.58 (m, 8H), 1.62–1.67 (m, 4H), 1.82–1.87 (m, 4H), 2.42 (br, 4H), 2.63 (br, 8H), 2.69–2.74 (m, 2H), 3.68 (s, 4H), 4.98 (s, 8H), 7.46 (t, 2H, *J* = 7.7 Hz), 7.53 (br, 2H), 7.95 (d, 4H, *J* = 7.8 Hz), 7.97 (s, 4H) ppm; ^13^C NMR (125 MHz, CDCl_3_): *δ* = 15.9, 16.4, 23.1, 23.4, 24.4, 28.5, 35.0, 46.1, 56.0, 60.7, 129.0, 129.1, 129.9, 131.7, 135.4, 136.8, 143.2, 143.5, 159.4 ppm; HRMS (ESI): *m*/*z* calcd for C_60_H_82_N_6_+H^+^: 887.6674 [M+H]^+^, found: 887.6679.

Compound **1** was prepared from **1-I** (114 mg, 0.14 mmol) and sodium borohydride (66 mg, 1.74 mmol) in 6 mL methanol/dichloromethane 2:1 (*v*/*v*). The reaction mixture was stirred for 21 h at room temperature. Yield 96% (111 mg, 0.14 mmol); m.p. 81 °C; ^1^H NMR (500 MHz, CDCl_3_): *δ* = 1.10 (t, 6H, *J* = 7.5 Hz), 1.15 (s, 18H), 1.25 (t, 12H, *J* = 7.5 Hz), 2.70 (q, 4H, *J* = 7.4 Hz), 2.81 (q, 8H, *J* = 7.4 Hz), 3.64 (s, 4H), 3.71 (s, 8H), 3.89 (s, 8H), 7.15–7.17 (m, 4H), 7.22–7.24 (m, 2H), 7.56 (s, 2H) ppm; ^13^C NMR (125 MHz, CDCl_3_): *δ* = 16.8, 17.0, 22.6 (2C), 28.9, 40.2, 47.6, 50.5, 55.1, 126.9, 127.1, 128.0, 134.3, 134.7, 140.7, 142.1, 142.2 ppm; HRMS (ESI): *m*/*z* calcd for C_54_H_82_N_6_+H^+^: 815.6674 [M+H]^+^, found: 815.6663.

Compound **2** was prepared from **2-I** (258 mg, 0.31 mmol) and sodium borohydride (141 mg, 3.73 mmol) in 9 mL methanol/dichloromethane 2:1 (*v*/*v*). The reaction mixture was stirred for 24 h at room temperature. Yield 75% (194 mg, 0.23 mmol); m.p. 74 °C; ^1^H NMR (500 MHz, CDCl_3_): *δ* = 1.10 (t, 6H, *J* = 7.4 Hz), 1.24 (t, 12H, *J* = 7.5 Hz), 1.35–1.43 (m, 4H), 1.50–1.57 (m, 4H), 1.64–1.71 (m, 4H), 1.79–1.85 (m, 4H), 2.68 (q, 4H, *J* = 7.5 Hz), 2.80 (q, 8H; *J* = 7.5 Hz), 3.17 (qui, 2H, *J* = 6.2 Hz), 3.65 (s, 4H), 3.71 (s, 8H), 3.89 (s, 8H), 7.15–7.17 (m, 4H), 7.22–7.26 (m, 2H), 7.54 (s, 2H) ppm; ^13^C NMR (125 MHz, CDCl_3_): *δ* = 16.8, 17.0, 22.6, 22.8, 24.2, 33.2, 46.7, 47.6, 55.1, 60.9, 126.9, 127.1, 128.0, 134.3, 134.5, 140.7, 142.0, 142.2 ppm; HRMS (ESI): *m*/*z* calcd for C_56_H_82_N_6_+H^+^: 839.6674 [M+H]^+^, found: 839.6676.

Compound **3** was prepared from **3-I** (369 mg, 0.43 mmol) and sodium borohydride (195 mg, 5.15 mmol) in 18 mL methanol/dichloromethane 2:1 (*v*/*v*). The reaction mixture was stirred for 24 h at room temperature. Yield 99% (370 mg, 0.43 mmol); m.p. 84 °C; ^1^H NMR (500 MHz, CDCl_3_): *δ* = 1.11–1.34 (m, 10H), 1.11 (t, 6H, *J* = 7.5 Hz), 1.25 (t, 12H, *J* = 7.5 Hz), 1.59–1.63 (m, 2H), 1.72–1.76 (m, 4H), 1.89–1.93 (m, 4H), 2.51–2.56 (m, 2H), 2.70 (q, 4H, *J* = 7.5 Hz), 2.82 (q, 8H, *J* = 7.5 Hz), 3.70 (s, 4H), 3.72 (s, 8H), 3.90 (s, 8H), 7.17–7.18 (m, 4H), 7.24–7.25 (m, 2H), 7.56 (s, 2H) ppm; ^13^C NMR (125 MHz, CDCl_3_): *δ* = 16.7, 16.9, 22.5, 22.7, 25.0, 26.1, 33.5, 45.2, 47.5, 55.0, 58.0, 126.8, 127.0, 127.9, 134.2, 134.6, 140.6, 141.9, 142.1 ppm; HRMS (ESI): *m*/*z* calcd for C_58_H_86_N_6_+H^+^: 867.6987 [M+H]^+^, found: 867.6967.

Compound **4** was prepared from **4-I** (179 mg, 0.20 mmol) and sodium borohydride (92 mg, 2.40 mmol) in 7.5 mL methanol/dichloromethane 2:1 (*v*/*v*). The reaction mixture was stirred for 24 h at room temperature. Yield 98% (176 mg, 0.19 mmol); m.p. 78 °C; ^1^H NMR (500 MHz, CDCl_3_): *δ* = 1.10 (t, 6H, *J* = 7.5 Hz), 1.24 (t, 12H, *J* = 7.5 Hz), 1.39–1.46 (m, 8H), 1.49–1.59 (m, 8H), 1.63–1.68 (m, 4H), 1.82–1.86 (m, 4H), 2.69 (q, 4H, *J* = 7.6 Hz), 2.69–2.74 (m, 2H), 2.80 (q, 8H, *J* = 7.6 Hz), 3.65 (s, 4H), 3.71 (s, 8H), 3.89 (s, 8H), 7.14–7.17 (m, 4H), 7.22–7.24 (m, 2H), 7.55 (s, 2H) ppm; ^13^C NMR (125 MHz, CDCl_3_): *δ* = 16.8, 17.1, 22.6, 22.8, 24.4, 28.5, 35.0, 46.0, 47.6, 55.1, 60.6, 126.9, 127.1, 128.0, 134.3, 134.7, 140.7, 142.0, 142.2 ppm; HRMS (ESI): *m*/*z* calcd for C_60_H_90_N_6_+H^+^: 895.7299 [M+H]^+^, found: 895.7283.

## Figures and Tables

**Table 1:** Association constants for the complexation of octyl *β*-d-glucopyranoside with compounds **1–4** (for further data, see Table 2).

Compound	X Unit of the Side-Arms	*K*_11_^a,b,d^ [m^−1^] NMR ^b^	*K*_11_^a,c,d^ [m^−1^] ITC ^c^
CDCl_3_ (NMR), CHCl_3_ (ITC)			
**1** **2** **3** **4**	*tert*-butylamino cyclopentylamino cyclohexylamino cycloheptylamino	7960 11,200 12,500 9800	7780 10,800 12,300 8840
CHCl_3_/H_2_O (0.035 M H_2_O; ITC)			
**3**	cyclohexylamino	--	22,650

^a^ 1:1 Receptor-substrate binding model. ^b^Average *K*_11_ values from multiple ^1^H NMR spectroscopic titrations in CDCl_3_ (293 K; evaluated on the basis of WinEQNMR [71] and SupraFit [72] programs). ^c^ Average *K*_11_ values from multiple microcalorimetric titrations in CHCl_3_ or CHCl_3_/H_2_O (0.035 M H_2_O) (293 K; evaluated on the basis of NanoAnalyze program). ^d^ Errors were estimated at <4%.

**Table 2:** Results of microcalorimetric titrations of compounds **1**–**4** with **βGlc** in dry CHCl_3_ and CHCl_3_/H_2_O (0.035 M H_2_O) at 20 °C ^a^.

Compound	*K*_11_ [M^−1^]	−∆*G* [kJ/mol]	−∆*H* [kJ/mol]	−T∆*S* [kJ/mol]	−∆*S* [J/mol K]
**1** ^b^	7780	21.8	98.7	76.8	262
**2** ^b^	10,800	22.6	103.8	81.2	277
**3** ^b^	12,300	23.0	98.6	75.6	258
**3** ^c^	22,650	24.4	102.2	77.8	265
**4** ^b^	8840	22.1	103.1	81.0	276

^a^ The errors of *K*_11_ are <4%, the errors of ∆*H* are <1% (standard deviations for a minimum of three replicated ITC titrations). ^b^ In dry CHCl_3_ at 20 °C. ^c^ In CHCl_3_/H_2_O (0.035 M H_2_O) at 20 °C.

## Data Availability

The data presented in this study are available on request from the corresponding author.

## Supplementary Materials

The following supporting information can be downloaded at: https://www.mdpi.com/article/10.3390/molecules27217630/s1. Description of the binding studies: ^1^H NMR titrations and microcalorimetric investigations (ITC). Figures S1 and S2: 1H NMR spectroscopic titrations (further examples). Figures S3 and S4: ITC binding studies (further examples). Figure S5: Molecular modelling studies (example). Figures S6 and S7: ROESY studies for **3**·**βGlc**. Figures S8–S15: 1H and 13C NMR spectra of compounds **1**–**4**. Figures S16–S23: 1H and 13C NMR spectra of compounds **1-I**–**4-I**. Figures S24–S31: 1H and 13C NMR spectra of compounds **7**–**10**. Table S1: 1H NMR titration of compound **3** with octyl-β-D-glucopyranoside **(βGlc**) in CDCl_3_.

## Sample Availability

Samples of all obtained compounds are available from the authors.
