# Supplementary material for: Cycloalkyl Groups as Building Blocks of Artificial Carbohydrate Receptors: Studies with Macrocycles Bearing Flexible Side-Arms
Source: Molecules. 2022 Nov 7;27(21):7630. doi: 10.3390/molecules27217630 (PMC9654292; doi:10.3390/molecules27217630)
Supplement: Supplementary file 1 [file molecules-27-07630-s001.zip › molecules-1957510-supplementary.pdf]

# Supplementary Materials

## Cycloalkyl groups as building blocks of artificial carbohydrate receptors: Studies with macrocycles bearing flexible side-arms

Betty Leibiger, Manuel Stapf and Monika Mazik*

Institut für Organische Chemie, Technische Universität Bergakademie Freiberg, Leipziger Straße 29, 09596 Freiberg, Germany, Tel.: 03731392389; Fax: 03731393170

e-mail: monika.mazik@chemie.tu-freiberg.de; https://tu-freiberg.de/fakultaet2/orgch

**1** Description of the binding studies

1.1 Description of the $^{1}H$ NMR titrations

1.2 Description of the microcalorimetric investigations (ITC)

**2** $^{1}H$ NMR spectroscopic titrations (further examples; Figures S1 and S2)

**3** ITC binding studies (further examples; Figures S3 and S4)

**4** Molecular modelling studies (example; Figure S5)

**5** ROESY studies for **3·βGlc** (Figures S6 and S7)

**6** $^{1}H$ and $^{13}C$ NMR spectra of compounds **1** – **4** (Figures S8-S15)

**7** $^{1}H$ and $^{13}C$ NMR spectra of compounds **1-I** – **4-I** (Figures S16-S23)

**8** $^{1}H$ and $^{13}C$ NMR spectra of compounds **7** – **10** (Figures S24-S31)

# 1 Description of the binding studies

## 1.1 Description of the $^{1}$H NMR titrations

The $^{1}$H NMR titrations were carried out in $CDCl_3$ at 20 °C [$CDCl_3$ was deacidified over basic aluminium oxide (Brockmann I) and stored over molecular sieve]. Stock solutions in $CDCl_3$ were prepared and homogenised for the respective receptor and sugar. These solutions and $CDCl_3$ were added together in a manner that the concentration of the receptor was kept constant and that of the sugar was varied. The receptor concentration was adjusted to about 1 mM to avoid self-aggregation. For the inverse titrations, the concentration of the sugar was kept constant and that of the receptor was varied accordingly. For each titration, 15 samples were prepared, thoroughly homogenised and the $^{1}$H NMR spectra were recorded. Table S1 shows a typical example of a titration table.

**Table S1:** $^{1}$H NMR titration of compound **3** with octyl-β-D-glucopyranoside (**βGlc**) in $CDCl_3$.

| | $V_{rec}$ [mL] | $c_{rec}$ [mM] | $V_{\beta Glc}$[mL] | $C_{\beta Glc}$ [mM] | $V_{solv}$ [mL] | ratio [**3**]/[**βGlc**] |
|---|---|---|---|---|---|---|
| 1 | 0.4 | 1.01 | 0.00 | 0.00 | 0.30 | 1:0.00 |
| 2 | 0.4 | 1.01 | 0.02 | 0.24 | 0.28 | 1:0.24 |
| 3 | 0.4 | 1.01 | 0.04 | 0.47 | 0.26 | 1:0.47 |
| 4 | 0.4 | 1.01 | 0.06 | 0.71 | 0.24 | 1:0.70 |
| 5 | 0.4 | 1.01 | 0.08 | 0.94 | 0.22 | 1:0.94 |
| 6 | 0.4 | 1.01 | 0.10 | 1.18 | 0.20 | 1:1.17 |
| 7 | 0.4 | 1.01 | 0.12 | 1.42 | 0.18 | 1:1.40 |
| 8 | 0.4 | 1.01 | 0.14 | 1.65 | 0.16 | 1:1.64 |
| 9 | 0.4 | 1.01 | 0.16 | 1.89 | 0.14 | 1:1.87 |
| 10 | 0.4 | 1.01 | 0.18 | 2.13 | 0.12 | 1:2.10 |
| 11 | 0.4 | 1.01 | 0.20 | 2.36 | 0.10 | 1:2.34 |
| 12 | 0.4 | 1.01 | 0.22 | 2.60 | 0.08 | 1:2.57 |
| 13 | 0.4 | 1.01 | 0.24 | 2.83 | 0.06 | 1:2.81 |
| 14 | 0.4 | 1.01 | 0.26 | 3.07 | 0.04 | 1:3.04 |
| 15 | 0.4 | 1.01 | 0.30 | 3.54 | 0.00 | 1:3.51 |

## 1.2 Description of the microcalorimetric investigations (ITC)

Isothermal titration calorimetry (ITC) was carried out in $CHCl_3$ [purity: p. a., distilled, deacified over basic aluminium oxide (Brockmann I) and stored over molecular sieve] at 20 °C on a Thermal Activity Monitor (TAM) 227 heat flow calorimeter from TA instruments (New Jersey, USA). The device is equipped as a twin system with a sample cell and a reference cell. Stock solutions of the receptor and the sugar were prepared. In the case of the measurement with water, the solutions were additionally degassed with ultrasound. The measuring cell (1 mL steel cell) was filled with 700 µL of the receptor stock solution, pre-tempered and constantly homogenised with the aid of a stirring motor. After a tempering phase of 5 h, the sugar solution was added to the receptor stock solution via a syringe with steel capillary over a total of 30 additions of 8 µL each (time span per addition: 30 min). At the end of the experiment, an electrical calibration is carried out with a net power of 50 µW.

# 2 $^{1}$H NMR spectroscopic titrations (further examples)

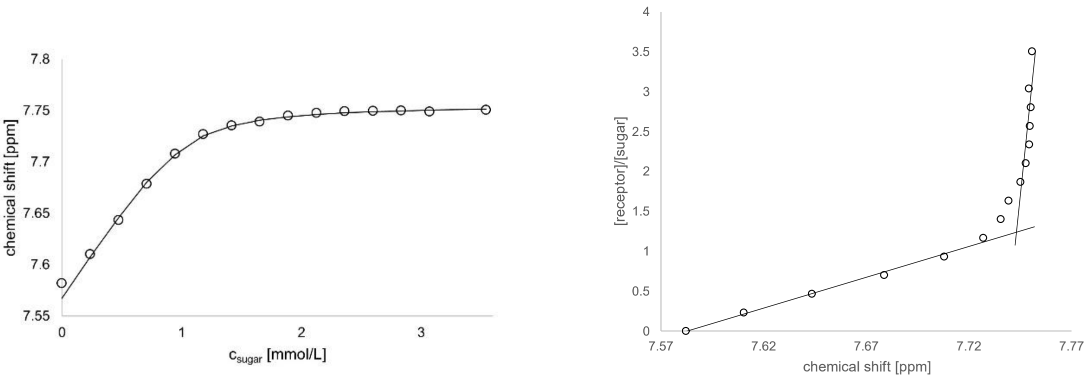


**Figure S1:** Plot of the experimental (circles) and calculated (line) chemical shift of the receptor signal CH[11] of compound **3** as a function of **βGlc** concentration (left) and the corresponding mole ratio plot (right).

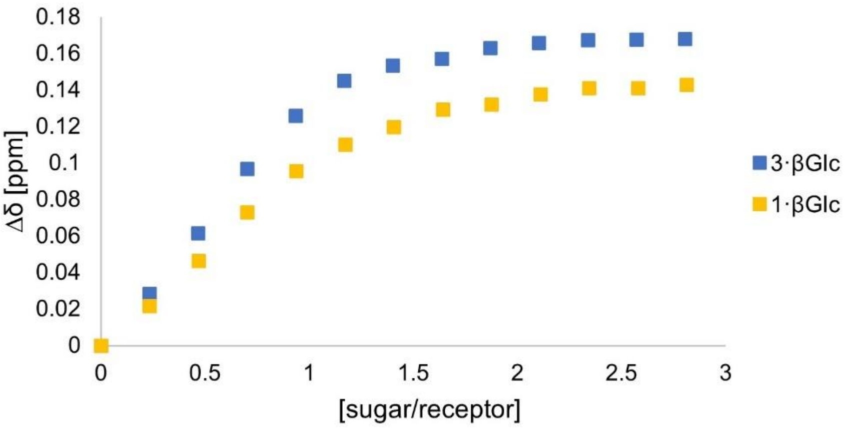


**Figure S2:** Changes in the chemical shift of the CH[11] signal of compound **1** and **3** for the $^{1}$H NMR titrations with·**βGlc**.

## 3 ITC binding studies (further examples)

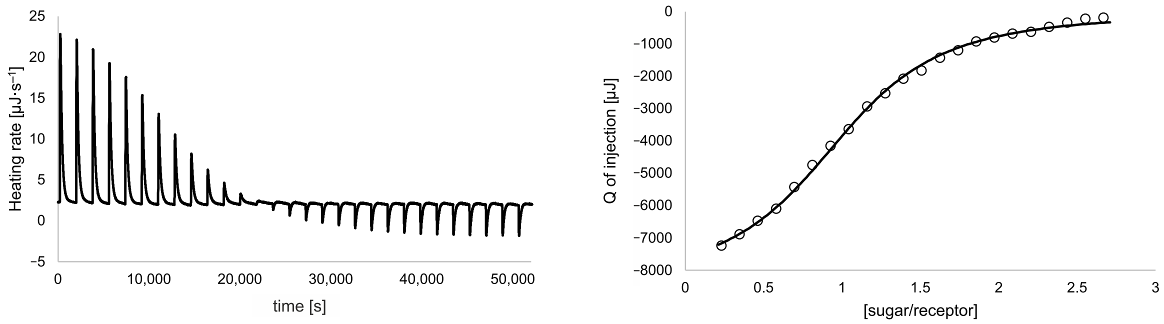


**Figure S3**: Exemplary ITC thermogram (left) and titration curve-fitting (right) for the titration of **1** with **βGlc** in $CHCl_3$ at 20 °C (heat of dilution was subtracted).

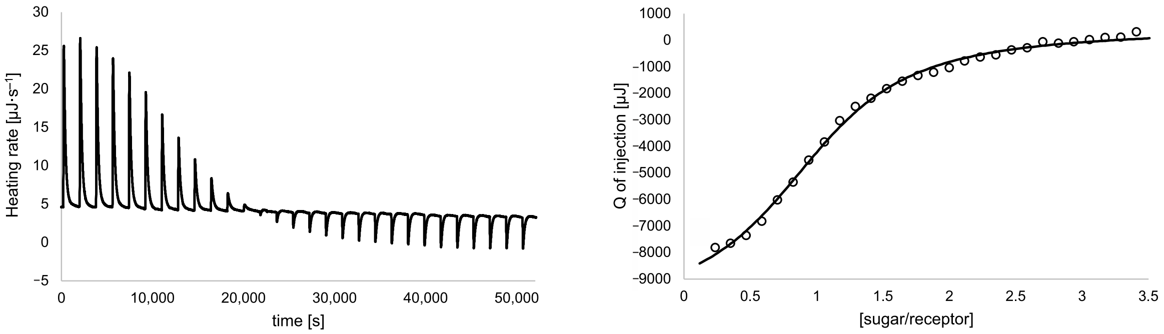


**Figure S4**: Exemplary ITC thermogram (left) and titration curve-fitting (right) for the titration of **4** with **βGlc** in $CHCl_3$ at 20 °C (heat of dilution was subtracted).

## 4 Molecular modelling calculations (example)

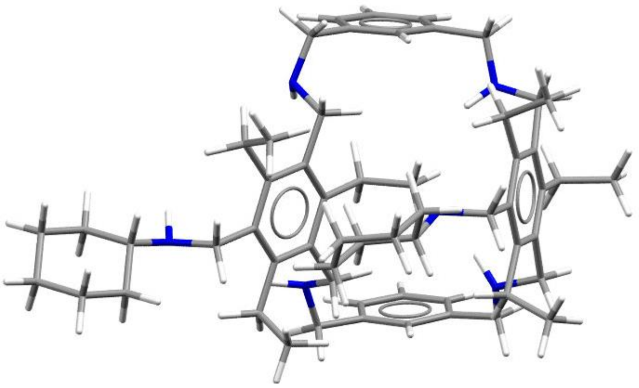

**Figure S5:** Energy-minimized structure of receptor **3**; C atoms: grey, N atoms: blue, H atoms: white (Maestro 11.0, OPLS_2005 force field, MCMM, 5000 steps).

## 5 ROESY studies for 3·βGlc

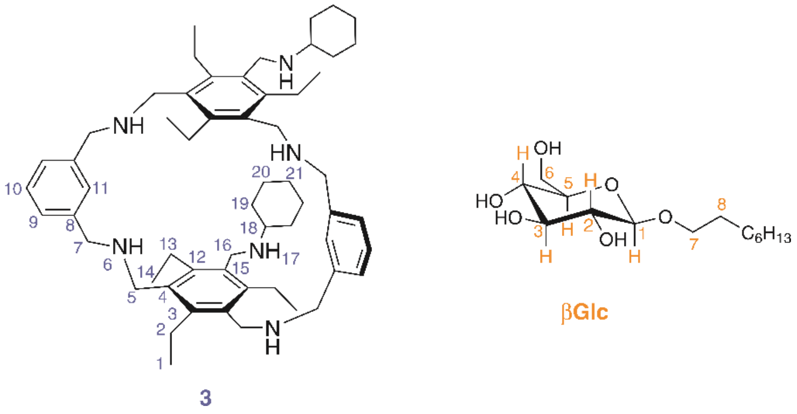


**Figure S6:** Signal assignments of **3** and **βGlc** for the evaluation of the ROESY spectrum (illustrated in Figure S7).

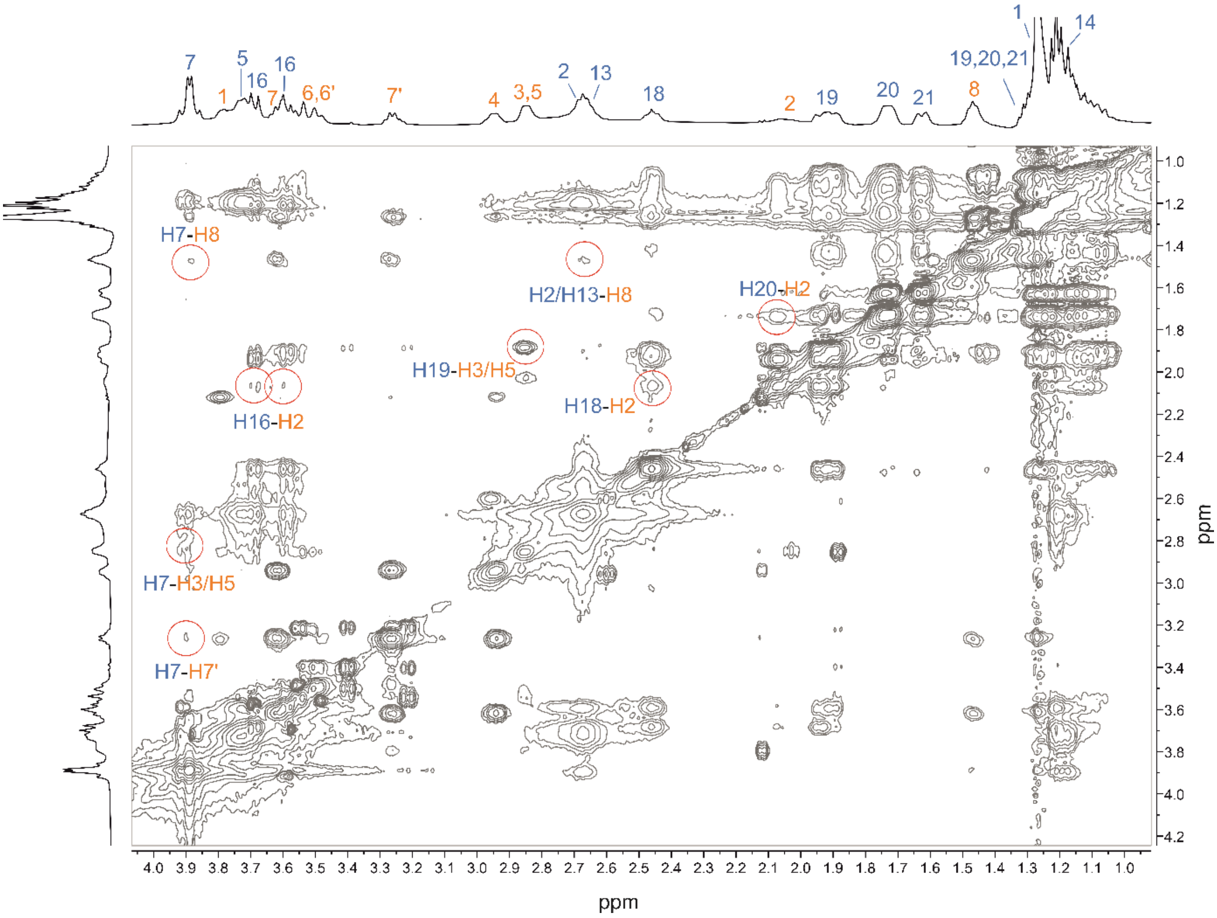


**Figure S7:** Excerpt of the ROESY spectrum of the complex **3·βGlc** in $CDCl_3$, the signals of the receptor are marked in blue, those of the carbohydrate in orange (assignment according to Figure S6); relevant couplings are marked exemplarily.

## 6 $^{1}H$ and $^{13}C$ NMR spectra of compounds 1 – 4

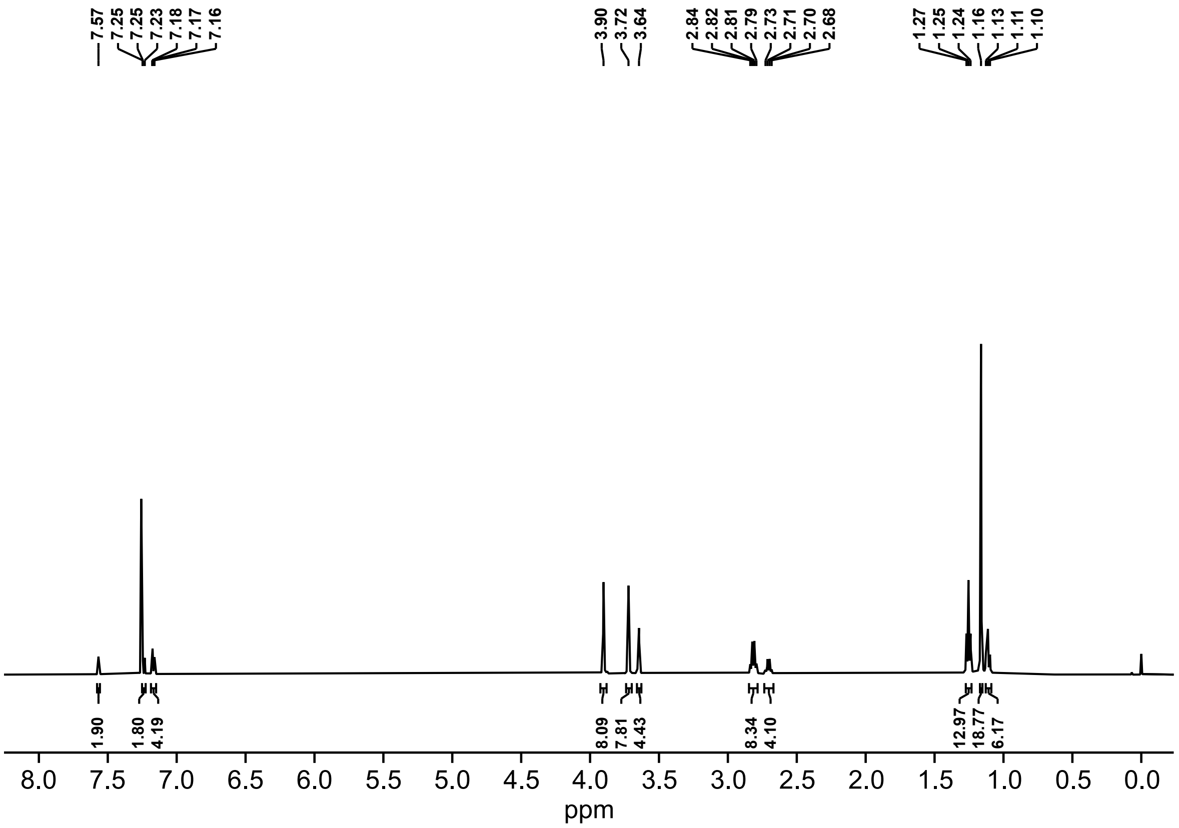

**Figure S8:** $^{1}H$ NMR spectrum of compound **1** in $CDCl_3$.

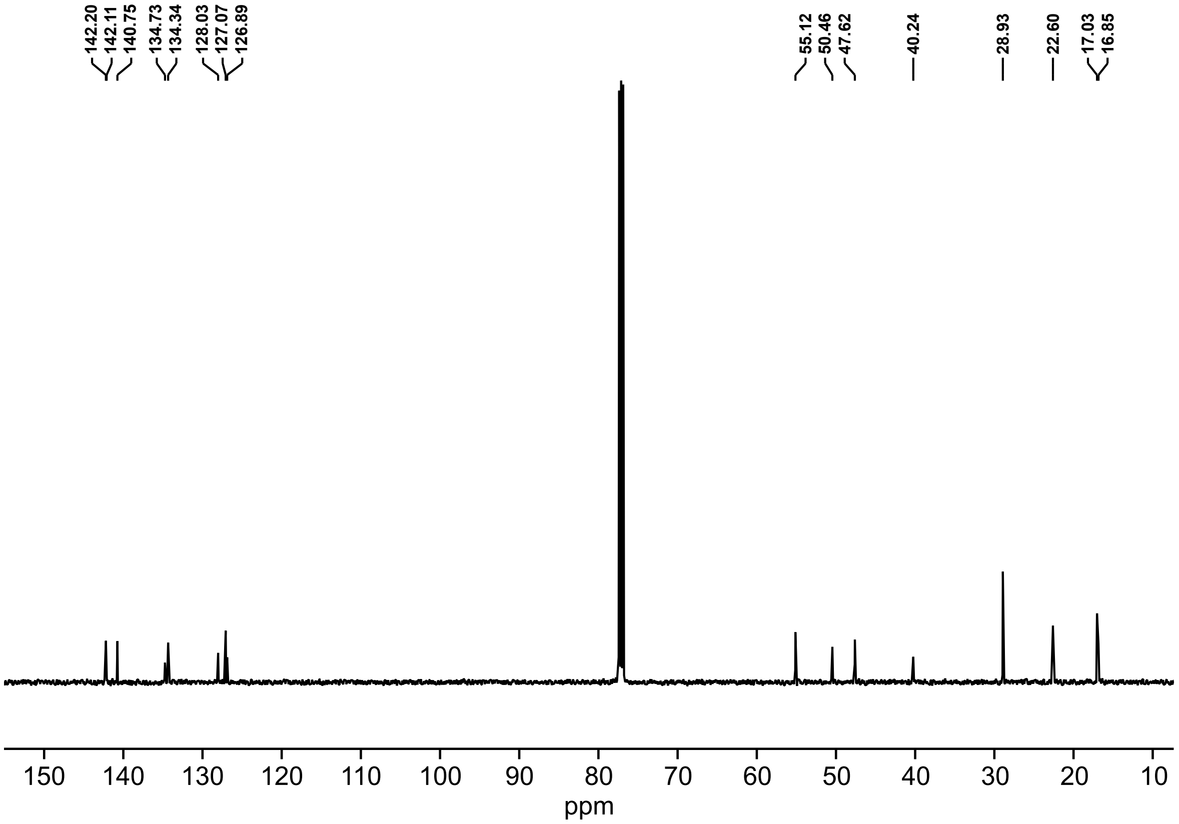

**Figure S9:** $^{13}C$ NMR spectrum of compound **1** in $CDCl_3$.

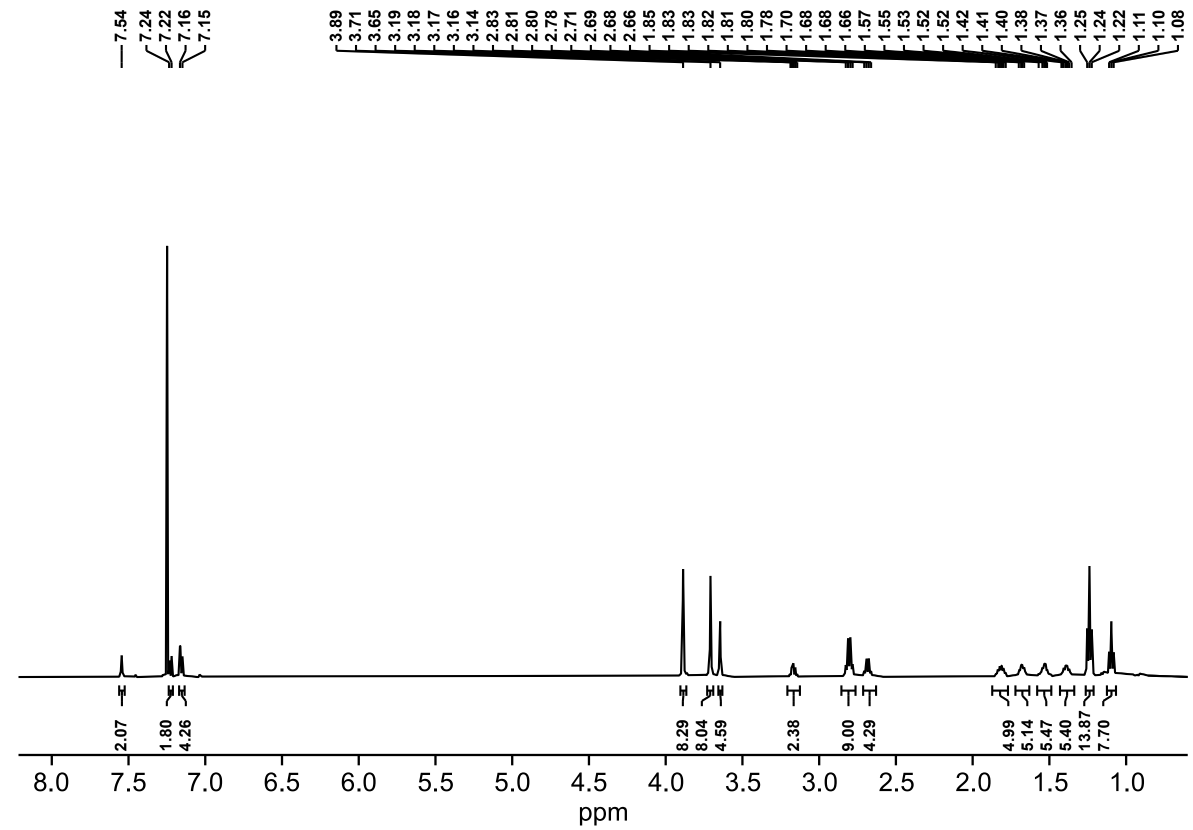


**Figure S10:** $^{1}$H NMR spectrum of compound **2** in $CDCl_3$.

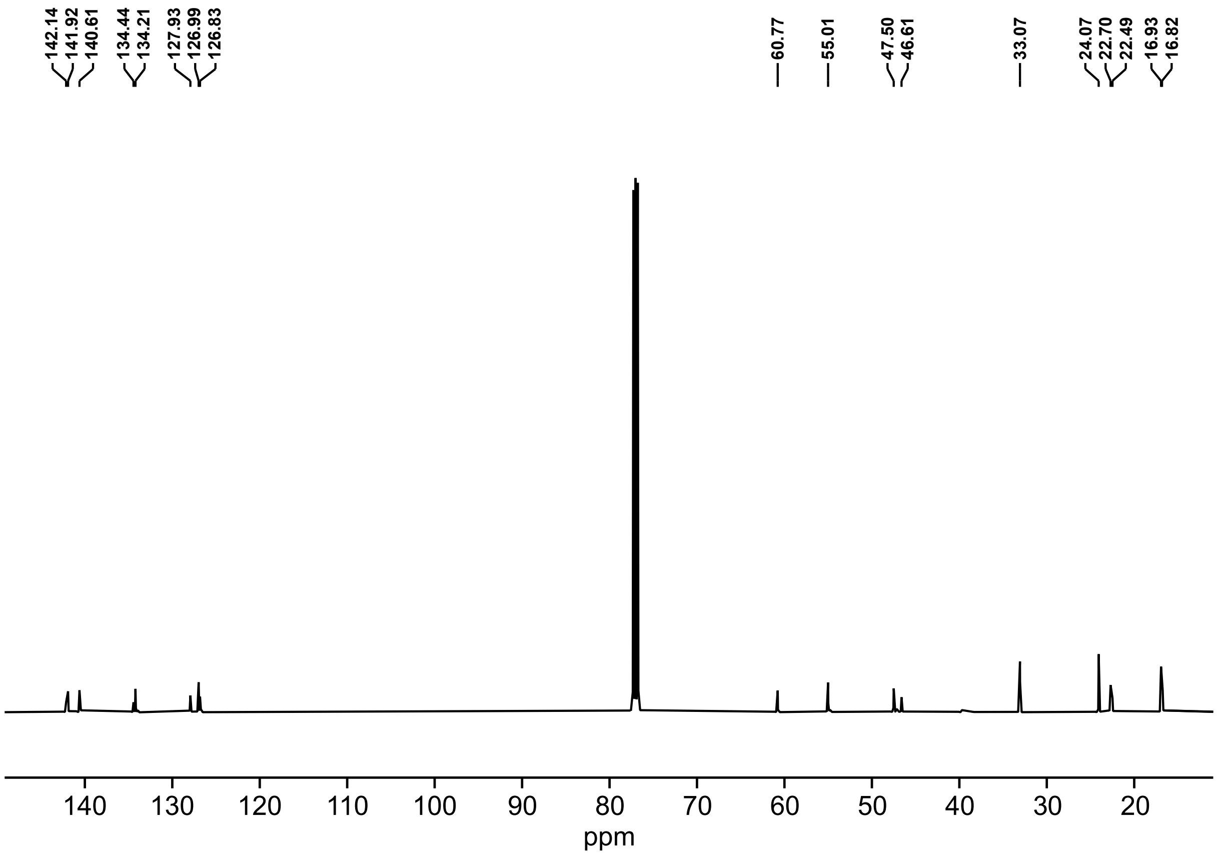


**Figure S11:** $^{13}$C NMR spectrum of compound **2** in $CDCl_3$.

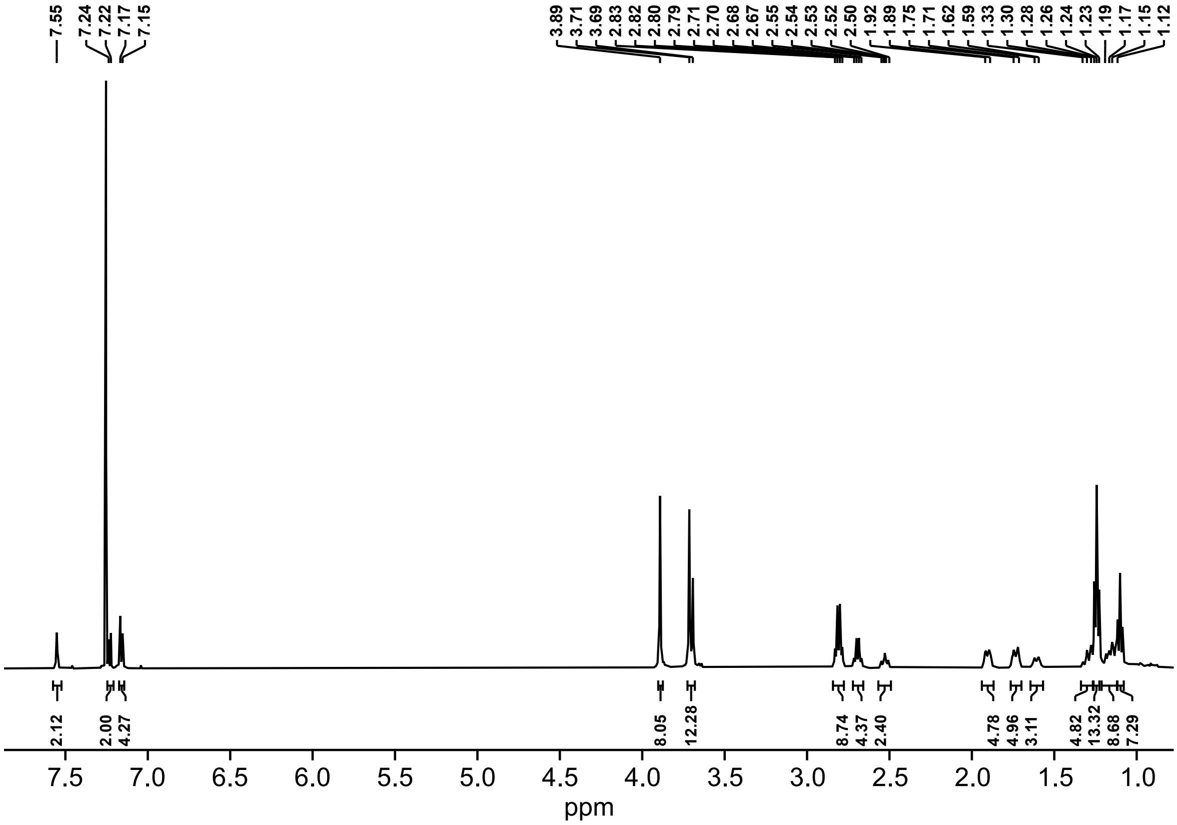


**Figure S12:** $^{1}$H NMR spectrum of compound **3** in $CDCl_3$.

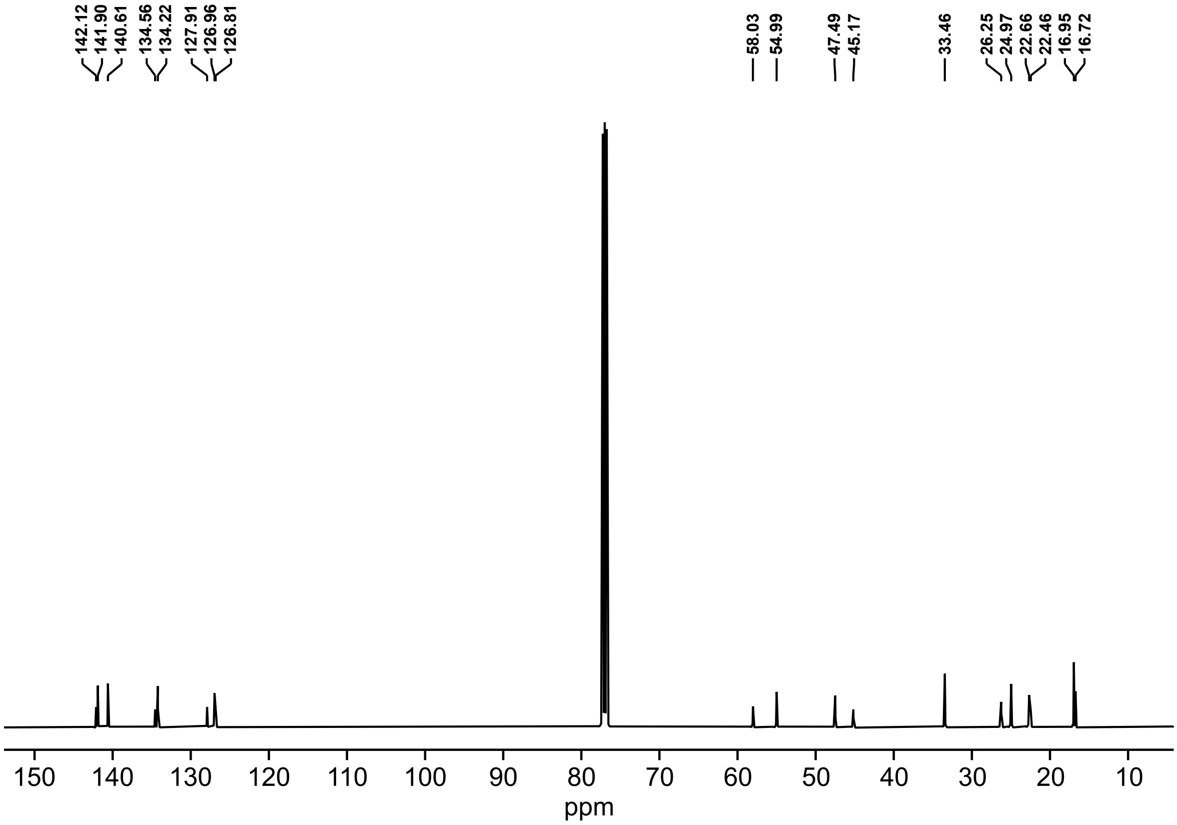


**Figure S13:** $^{13}$C NMR spectrum of compound **3** in $CDCl_3$.

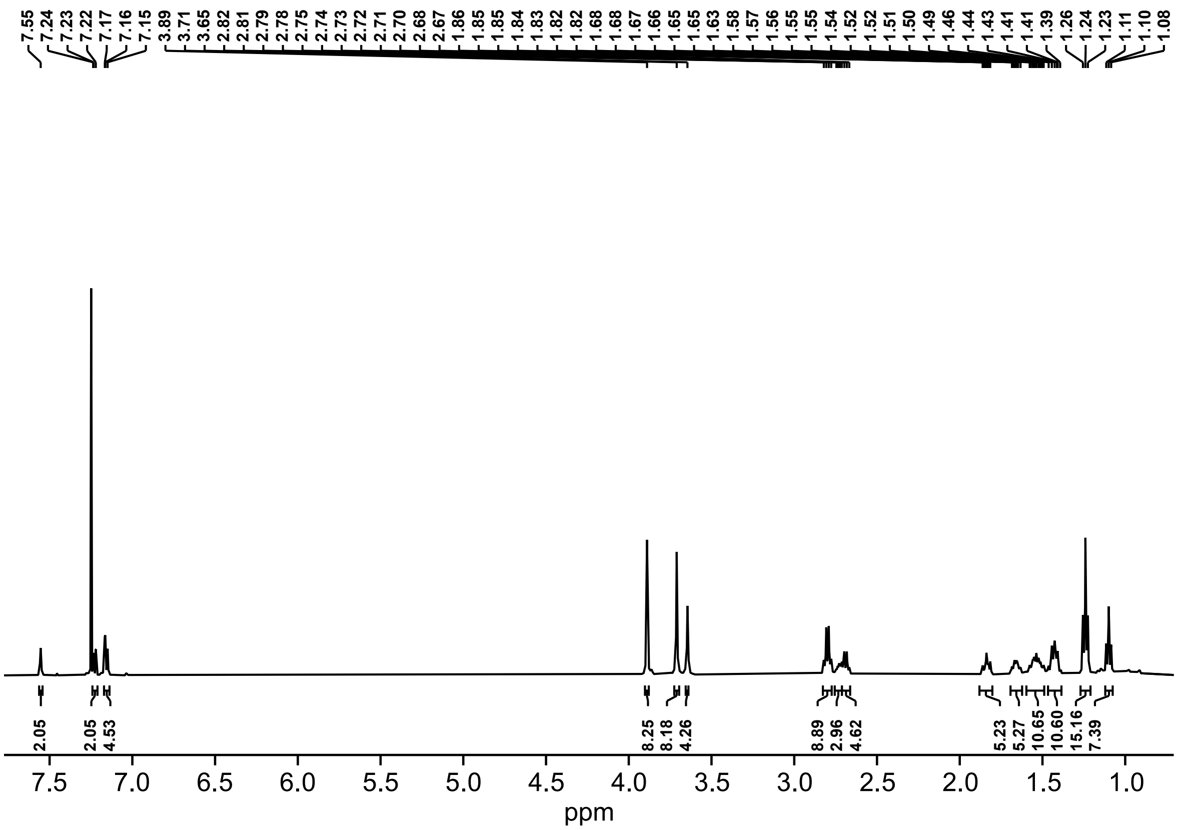

**Figure S14:** $^{1}H$ NMR spectrum of compound **4** in $CDCl_3$.

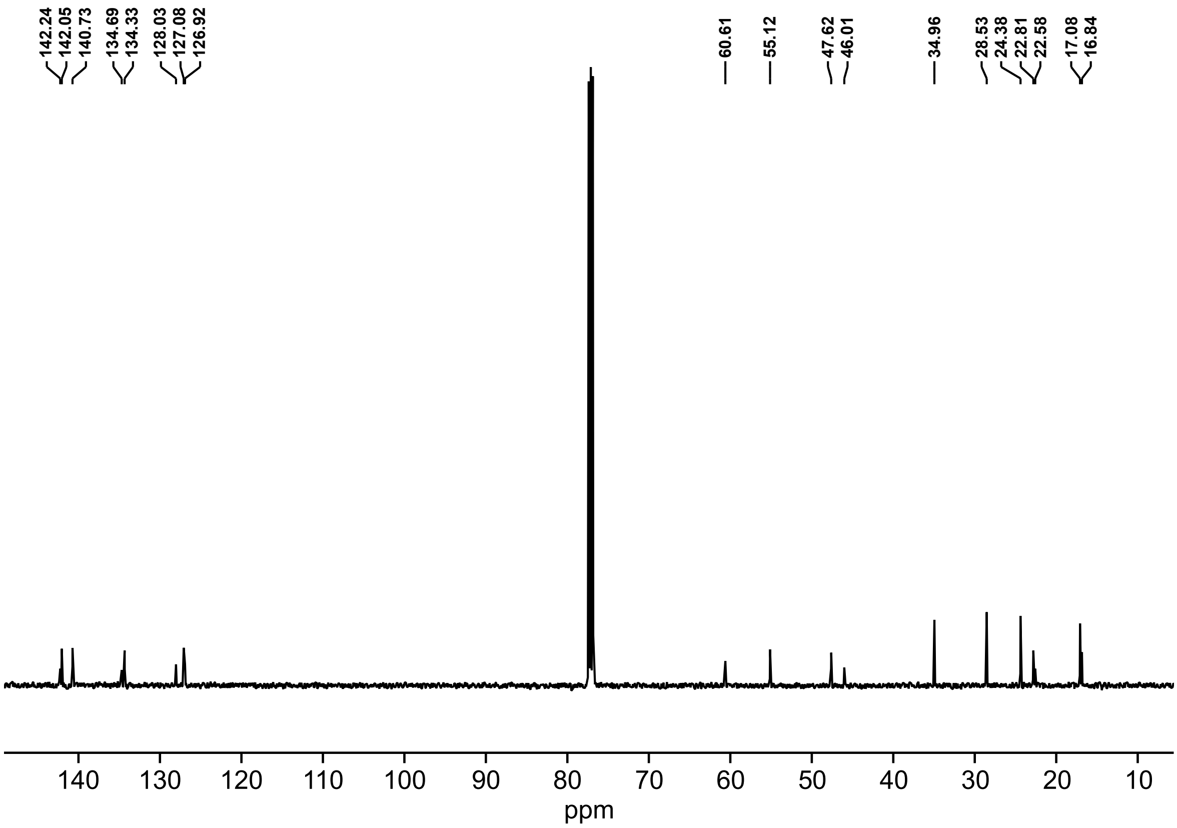

**Figure S15:** $^{13}C$ NMR spectrum of compound **4** in $CDCl_3$.

## 7 $^1$H and $^{13}$C NMR spectra of compounds 1-I – 4-I

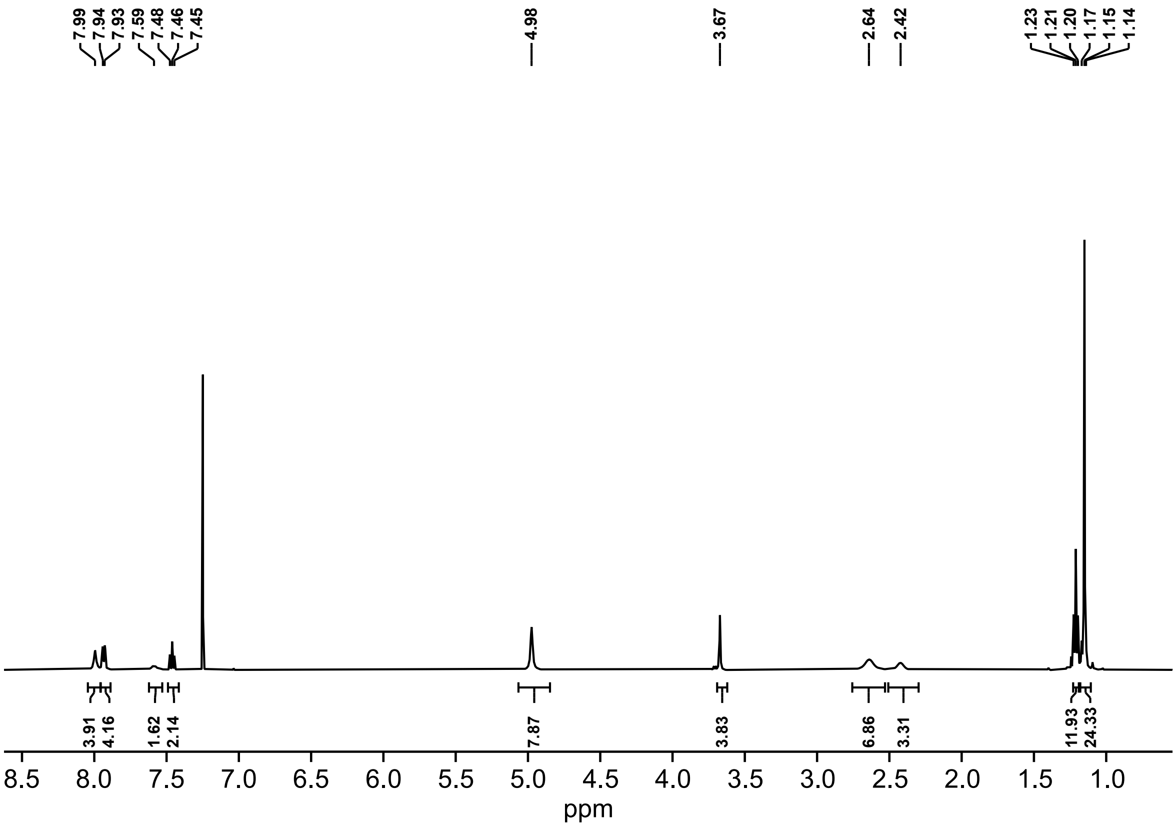


**Figure S16:** $^1$H NMR spectrum of compound **1-I** in $CDCl_3$.

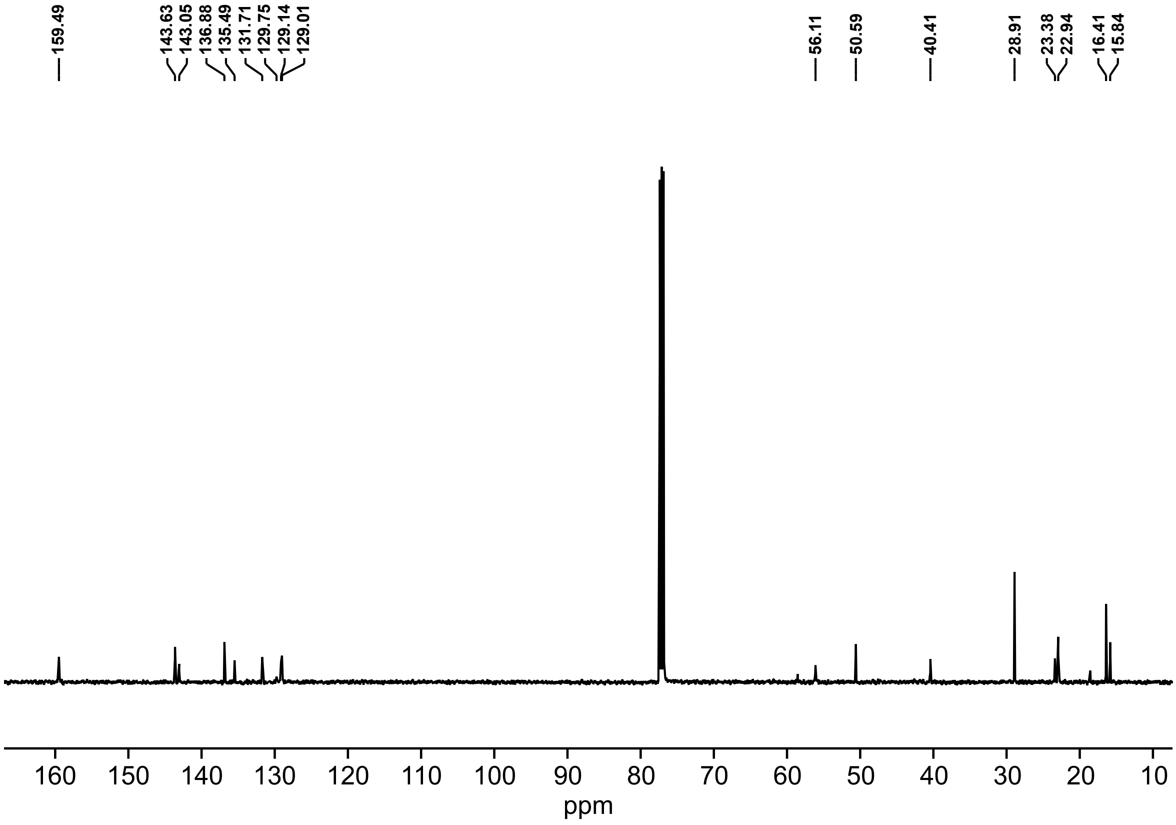


**Figure S17:** $^{13}$C NMR spectrum of compound **1-I** in $CDCl_3$.

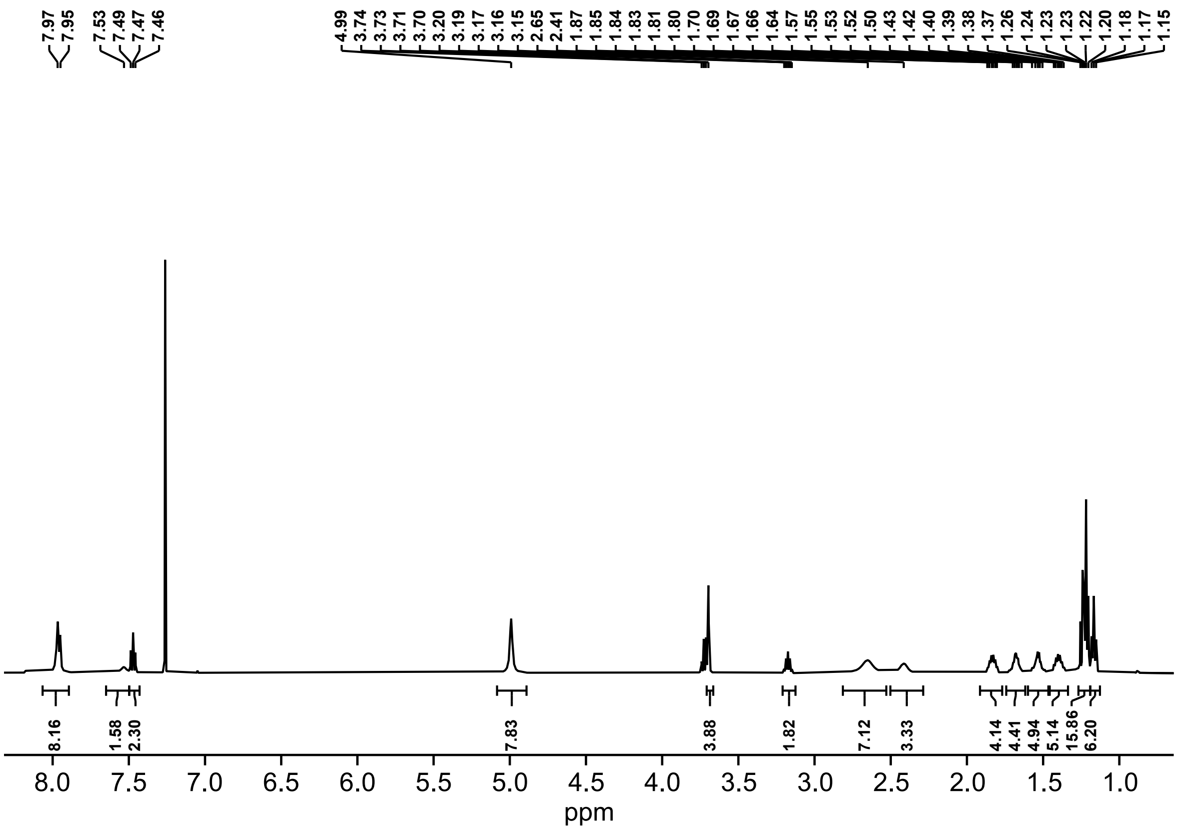

**Figure S18:** $^{1}$H NMR spectrum of compound **2-I** in $CDCl_3$.

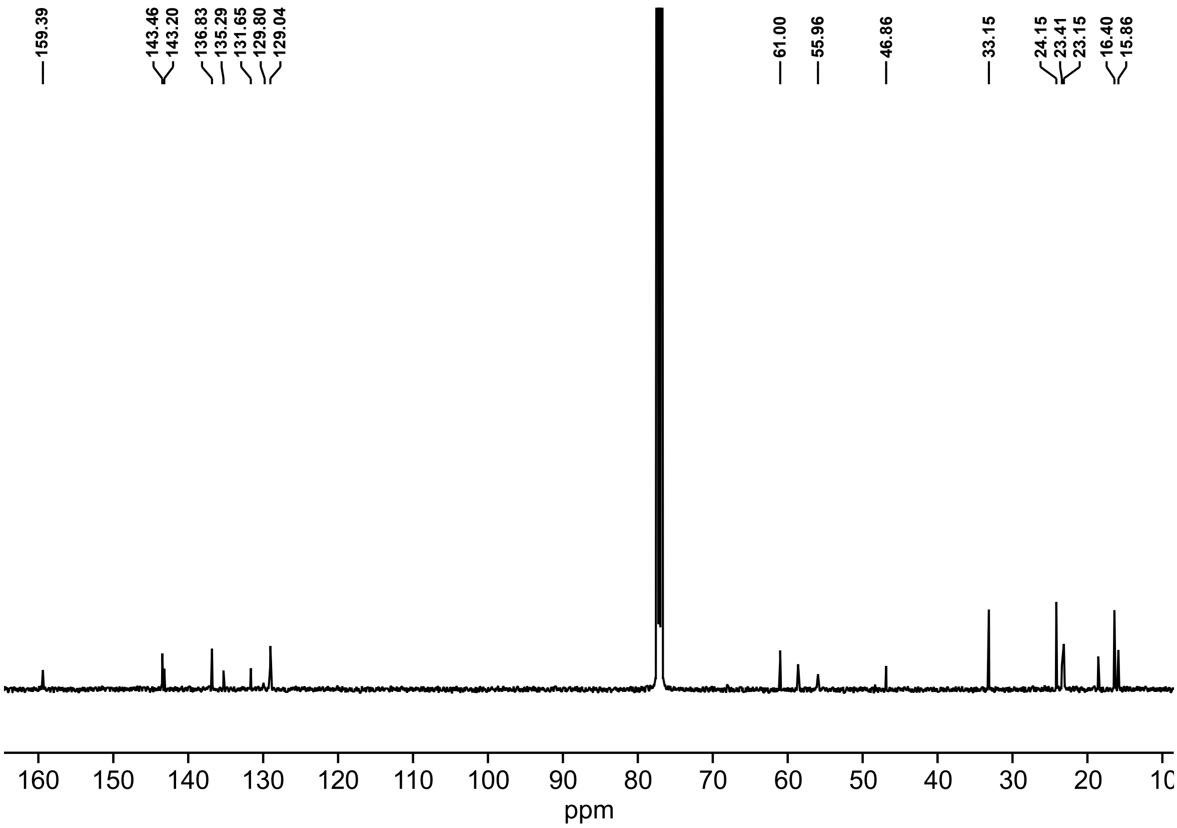

**Figure S19:** $^{13}$C NMR spectrum of compound **2-I** in $CDCl_3$.

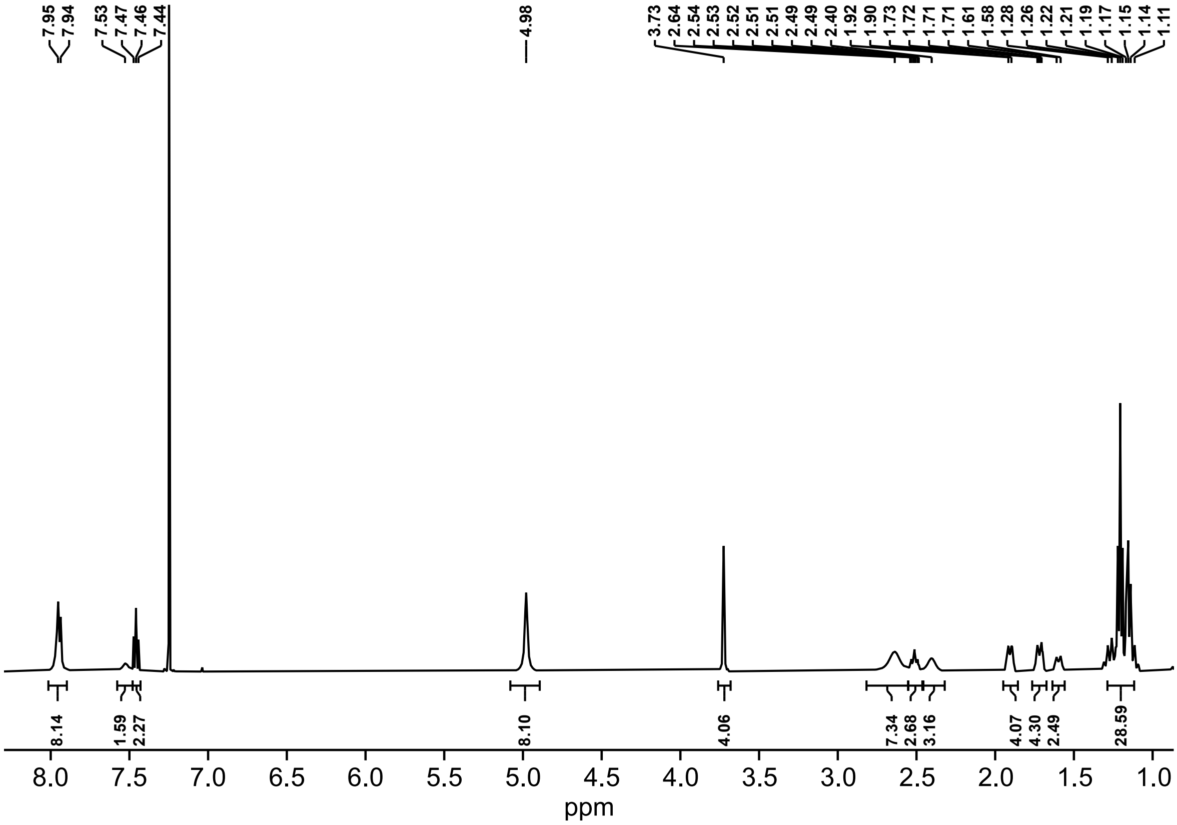


**Figure S20:** $^{1}$H NMR spectrum of compound **3-I** in $CDCl_3$.

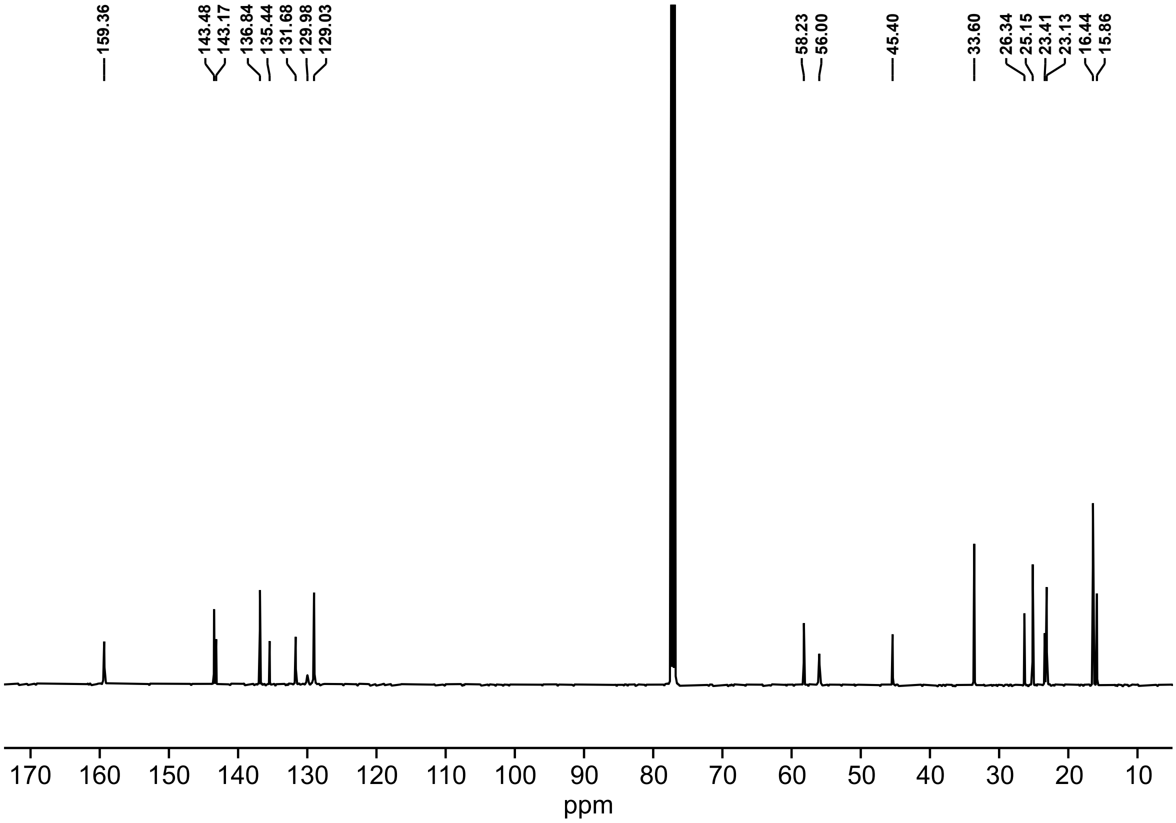


**Figure S21:** $^{13}$C NMR spectrum of compound **3-I** in $CDCl_3$.

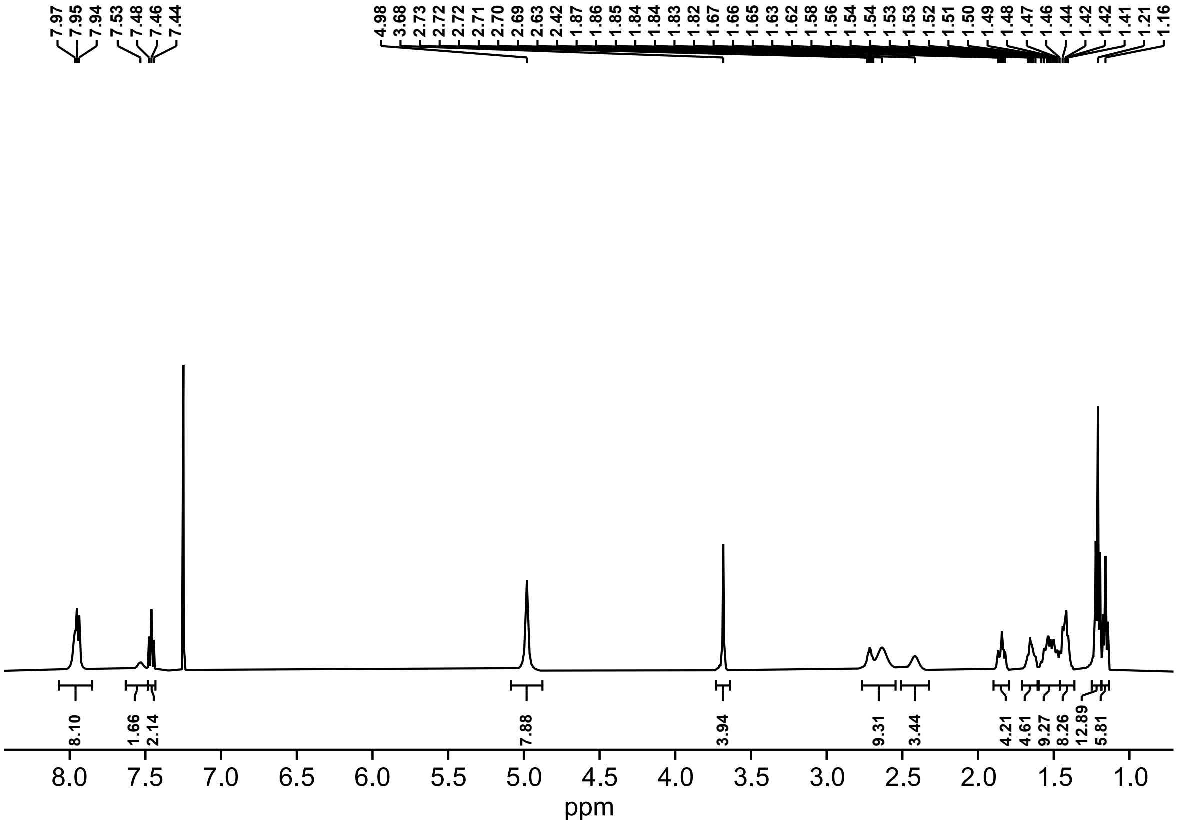

**Figure S22:** $^{1}$H NMR spectrum of compound **4-I** in $CDCl_3$.

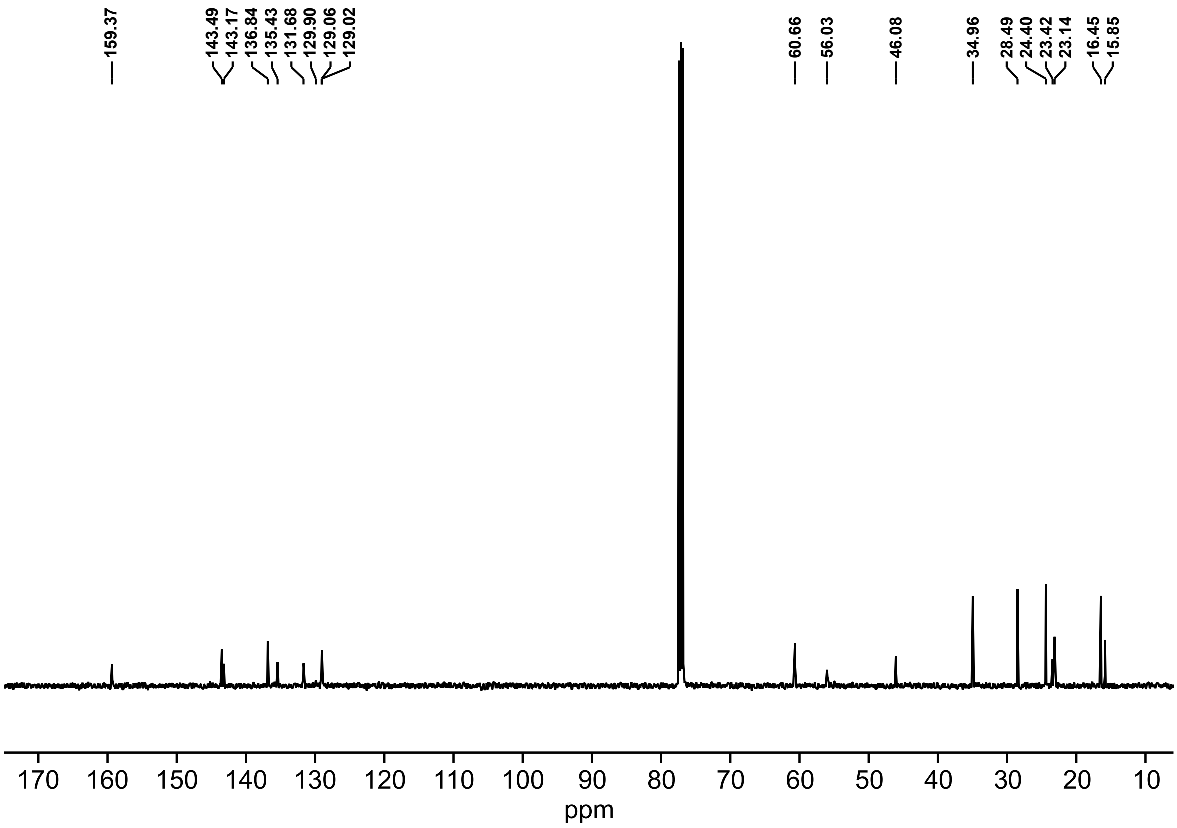

**Figure S23:** $^{13}$C NMR spectrum of compound **4-I** in $CDCl_3$.

## 8 $^{1}$H and $^{13}$C NMR spectra of compounds 7 – 10

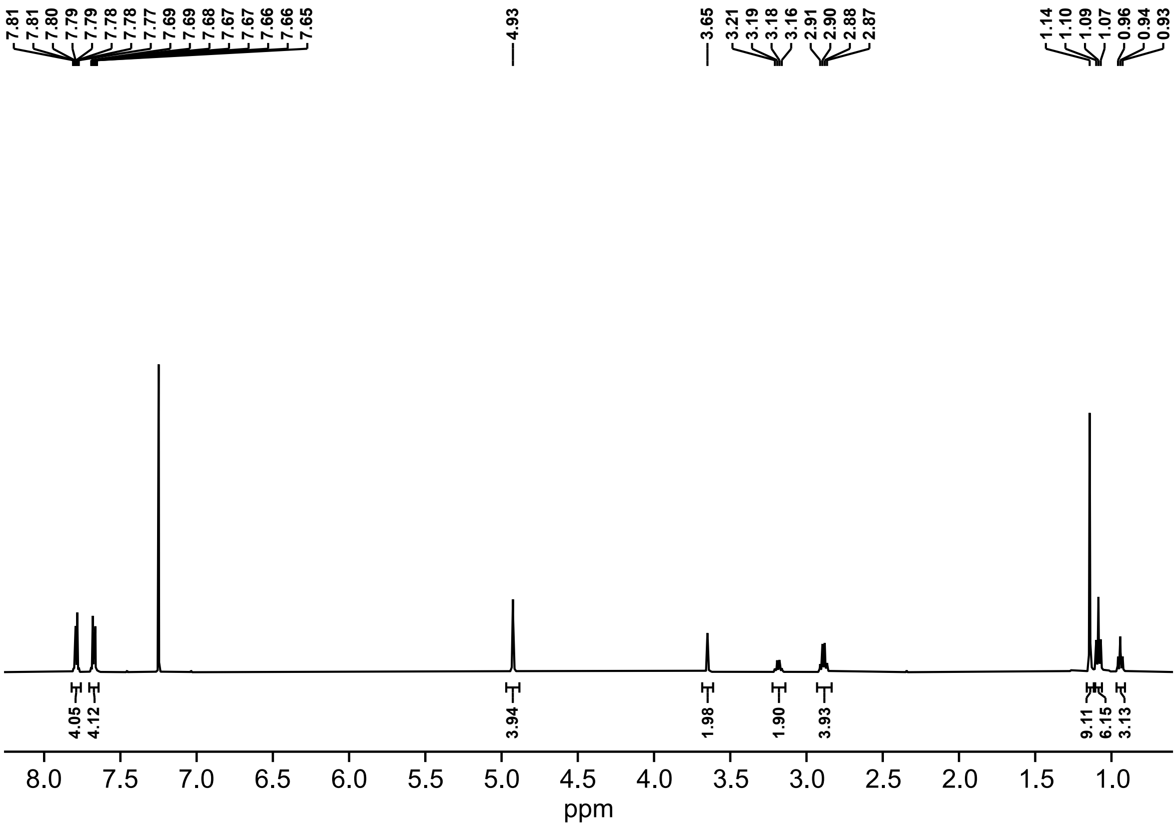


**Figure S24:** $^{1}$H NMR spectrum of compound **7** in $CDCl_3$.

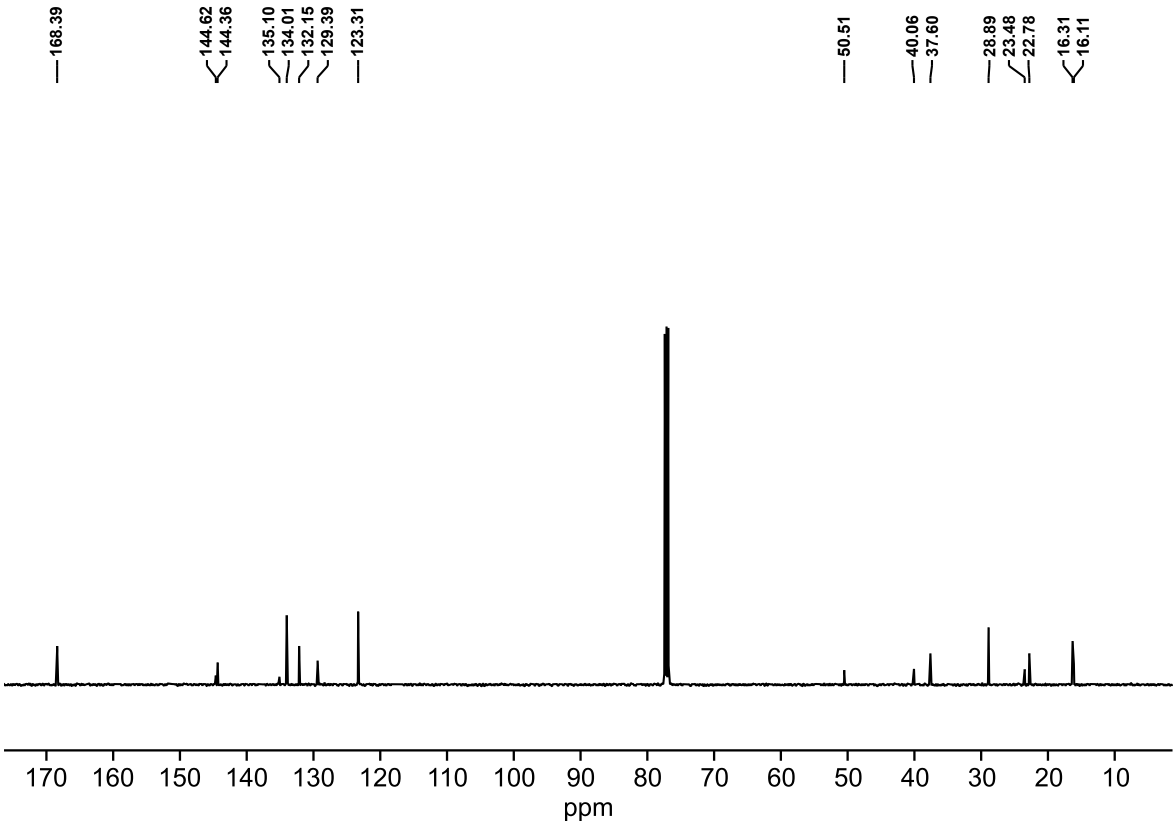


**Figure S25:** $^{13}$C NMR spectrum of compound **7** in $CDCl_3$.

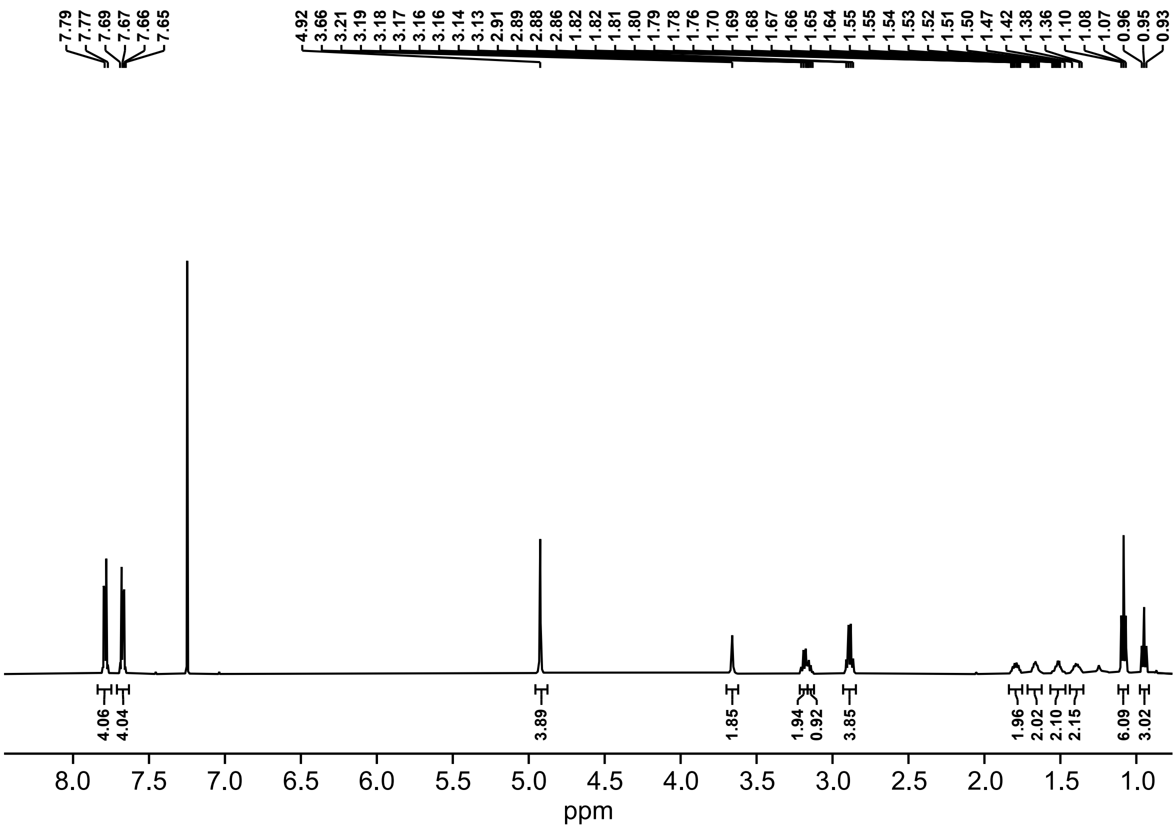


**Figure S26:** $^{1}H$ NMR spectrum of compound **8** in $CDCl_3$.

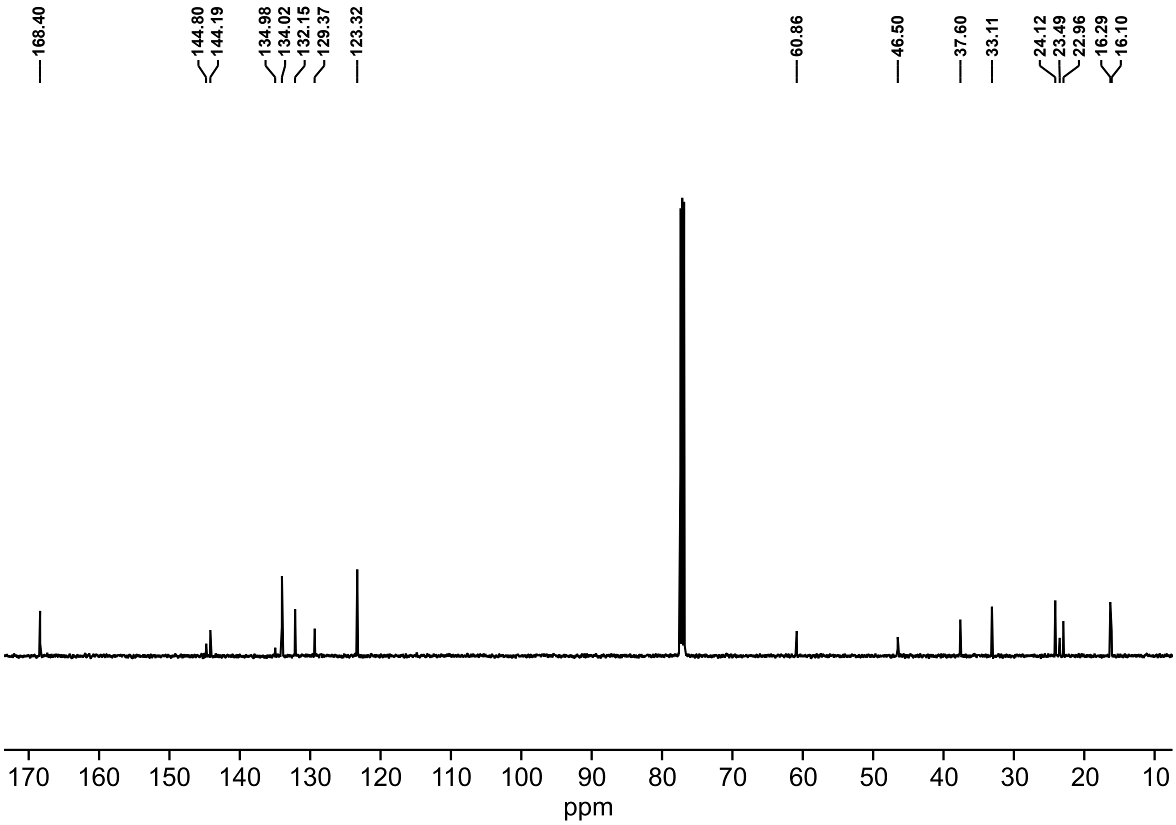


**Figure S27:** $^{13}C$ NMR spectrum of compound **8** in $CDCl_3$.

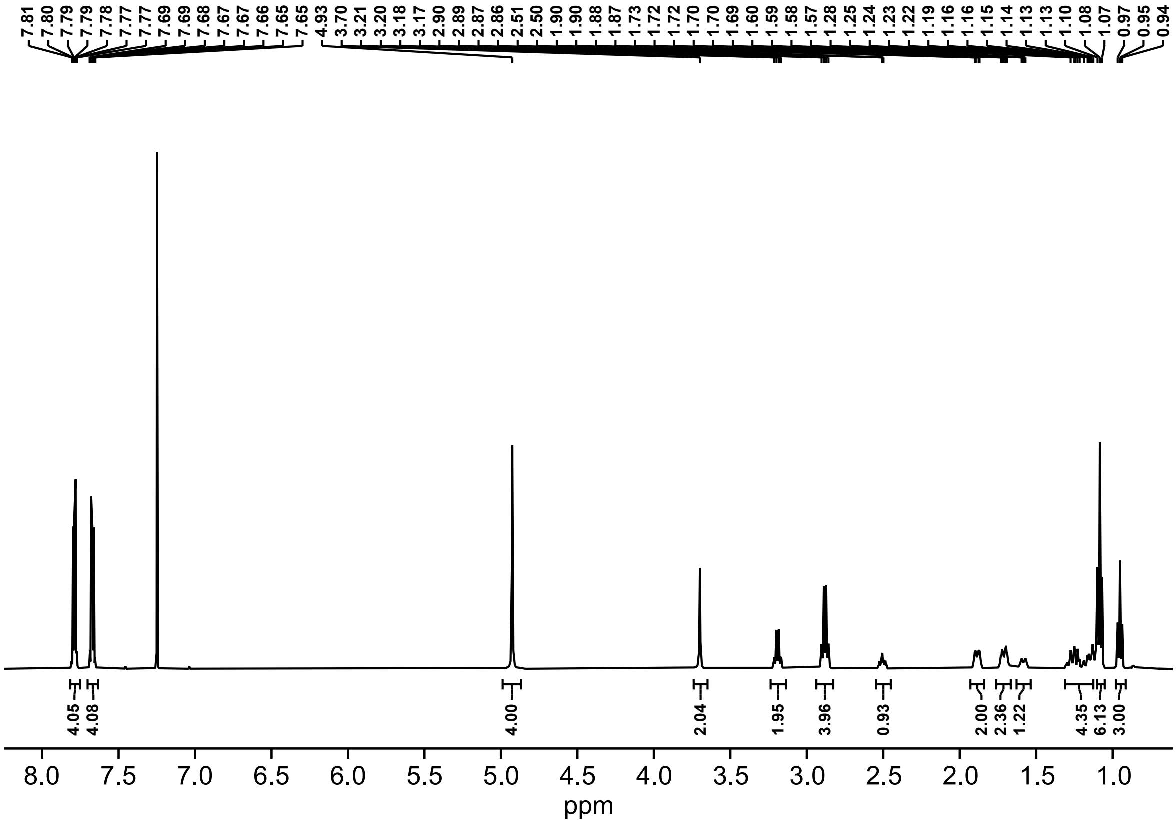


**Figure S28:** $^{1}$H NMR spectrum of compound **9** in $CDCl_3$.

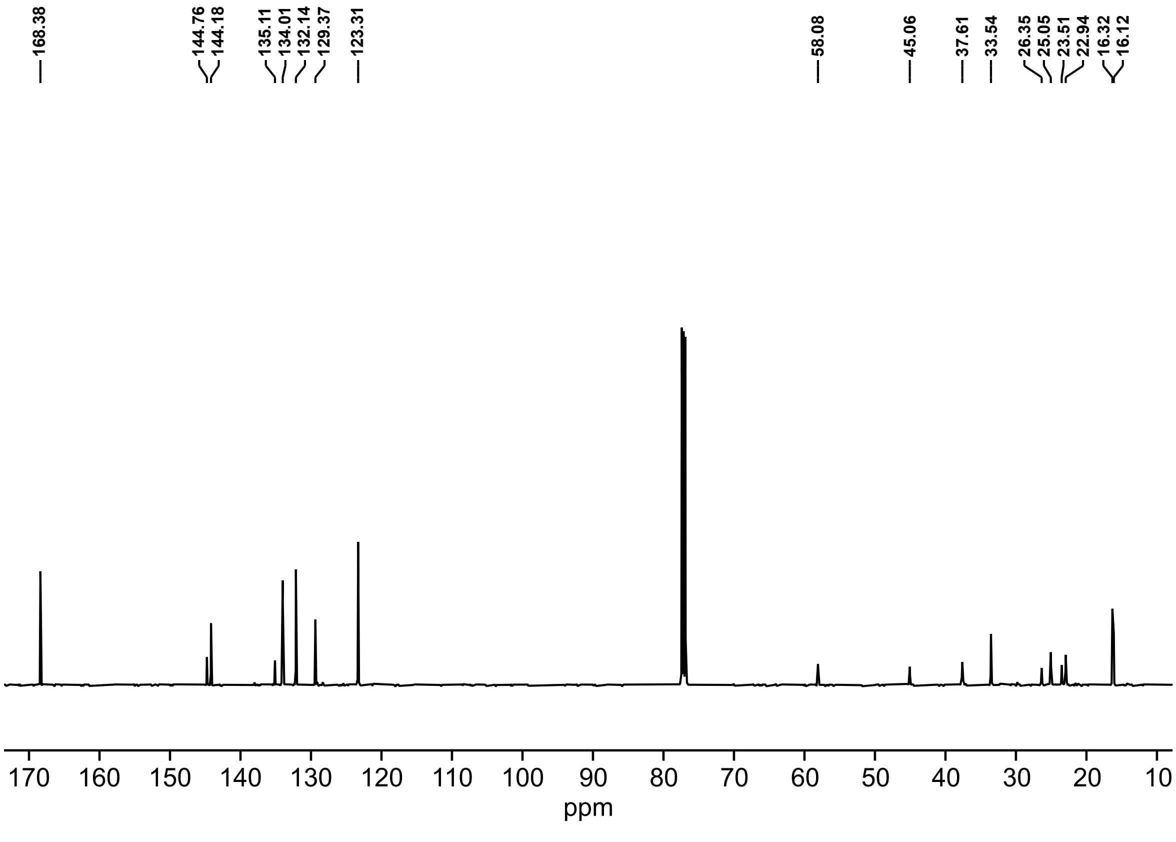


**Figure S29:** $^{13}$C NMR spectrum of compound **9** in $CDCl_3$.

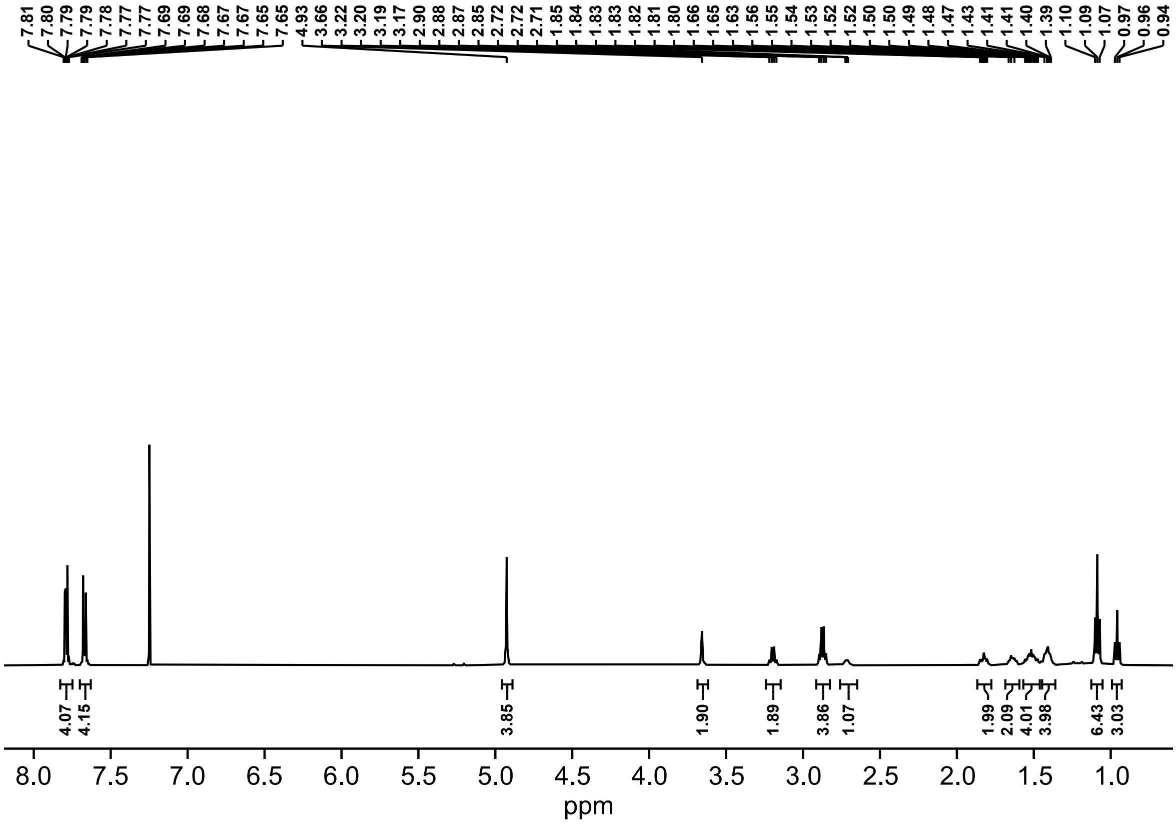


**Figure S30:** $^1$H NMR spectrum of compound **10** in $CDCl_3$.

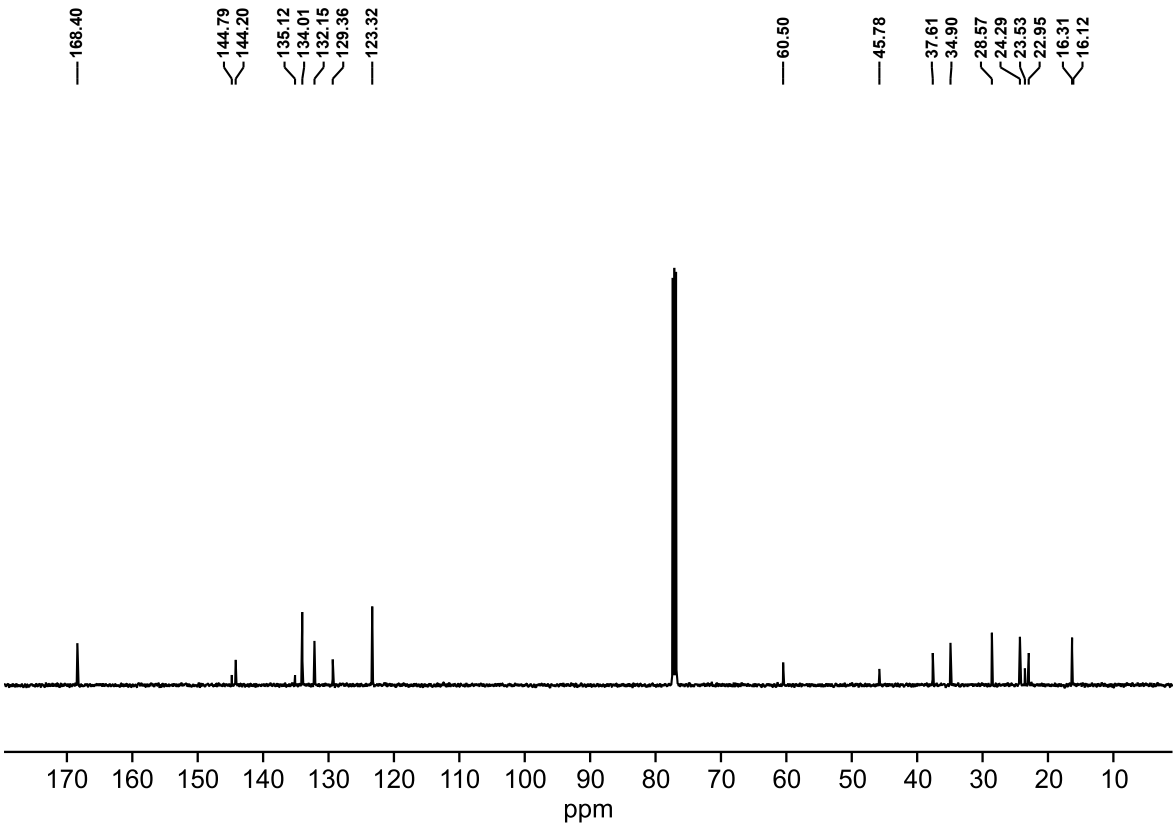


**Figure S31:** $^{13}$C NMR spectrum of compound **10** in $CDCl_3$.
